# Peritubular myoid cells of the testis produce monocyte chemotactic protein 1 upon direct exposure to Mono-(2-Ethylhexyl) phthalate through the IL-1 signaling pathway

**DOI:** 10.1016/j.tox.2025.154118

**Published:** 2025-03-13

**Authors:** Narayan Acharya, John H. Richburg

**Affiliations:** Division of Pharmacology and Toxicology, College of Pharmacy, University of Texas at Austin, Austin, TX 78712, USA

**Keywords:** MCP-1, PTMCs, Rats, Testis, Chemokine, IL-1 signaling

## Abstract

Mono-(2-ethylhexyl) phthalate (MEHP) is a metabolite of the diester parent compound Di(2-ethylhexyl) phthalate (DEHP), a widespread environmental toxicant known for its harmful effects on Sertoli cells and the subsequent loss of germ cells through apoptosis in postnatal animals. Peritubular myoid cells (PTMCs) produce various signaling factors, including the chemokine monocyte chemotactic protein 1 (MCP-1); however, the MEHP exposure-induced BTB disruption followed by MCP-1 secretion by PTMCs, the recruitment, and activation of macrophages as well as molecular mechanisms that initiate the secretion in the testis has yet to be closely examined. In this study, we demonstrate for the first time that PTMCs generate MCP-1 via the interleukin-1 signaling pathway upon MEHP exposure. Primary PTMCs isolated from the testis of peripubertal rats were cultured and exposed to 100 μM and 200 μM MEHP. Total RNA was used for bulk RNA sequencing, qRT-PCR, and protein lysates for proteomic analysis. Testis and their interstitial fluid (IF) were obtained from MEHP-exposed animals to evaluate the levels of pro-inflammatory cytokines and chemokines in IF through a multiplex assay and in tissue sections through immunofluorescence studies. The RNA sequencing data show significant enrichment of the interleukin-1 signaling pathway after MEHP (200 μM) exposure for 48 hours. This finding is further supported by the qRT-PCR results for select genes associated with the IL-1 signaling pathway, highlighting the crucial role of this pathway in the response of PTMCs to MEHP exposure. In summary, MEHP exposure stimulates MCP-1 production by PTMCs, and mechanistically, the IL-1 signal transduction pathway governs this response.

## 1. Introduction

Mono-(2-ethylhexyl) phthalate (MEHP), a prominent metabolite of Di(2-ethylhexyl) phthalate (DEHP), has been detected in maternal blood samples from pregnant Japanese women and urine samples from women and children in Taiwan, highlighting the widespread potential for human health risks (Araki et al., 2014; Su et al., 2014). The mechanisms underlying DEHP’s male reproductive toxicity are influenced by the age or life stage of exposure and involve oxidative stress, apoptosis of testicular cells, disruption of androgen synthesis, and alterations in the hypothalamic-pituitary-gonadal axis (Lin et al., 2023). Animal studies suggest that exposure to DEHP or MEHP hinders the proliferation and differentiation of testicular interstitial cells, germ cells, and Sertoli cells (Maekawa et al., 1996; Schmid et al., 2018; Zhou et al., 2019), while the biological responses at the gene and protein levels of peritubular myoid cells (PTMCs) are not yet fully understood.

Anatomically, PTMCs surround the outermost layer of cells in the seminiferous tubules (STs) and interact with the cells on either side of the ST through the secretion of various biological substances. For instance, the chemokine monocyte chemotactic protein 1 (MCP-1) produced by PTMCs contributes to the regeneration of stem and progenitor Leydig cells (Zhan et al., 2020). Additionally, glial cell line-derived neurotrophic factor (GDNF) secreted by PTMCs promotes the renewal of spermatogonial stem cells (SSC) (Chen et al., 2016, 2014; Spinnler et al., 2010). Therefore, the role of PTMCs in maintaining the SSC niche deserves further investigation.

Leukocyte migration, a vital component of the inflammatory response, is mainly regulated by chemokines. Chemokines are 8–10 kD chemotactic proteins that can be categorized into four families. Among the two well-characterized chemokine families, CXC or α-chemokines primarily attract neutrophils, while CC or β-chemokines attract various leukocytes, including monocytes and macrophages, with differing affinities. MCP-1 is a member of the β-chemokine family (CC chemokine) that promotes the infiltration of monocytes and macrophages into tissues (Luster, 1998). Several studies, including one from our group, have confirmed that PTMCs produce MCP-1 (Aubry et al., 2000; Mayerhofer, 2013; Murphy et al., 2014). Furthermore, a sustained increase in MCP-1 has been observed for up to 14 days in a mouse model of pneumopathy (Inoshima et al., 2004), while a similar study in Fisher 344 rats reported a significant rise in MCP-1 mRNA levels between days 3 and 21 (Zhang et al., 1994).

Previously, we reported that PTMC produces MCP-1 in response to MEHP exposure *in vivo* (Murphy et al., 2014). In contrast, another *in vitro* study indicated that MCP-1 mRNA and protein levels increase in PTMCs when exposed to Interleukin-1β, Tumor Necrosis Factor-alpha (TNF-α), and lipopolysaccharides (LPS) (Aubry et al., 2000), suggesting various pathways for MCP-1 production by PTMCs.

In a recent publication, we noted that exposure to the BTB-disrupting agent Cadmium chloride (CdCl_2_) leads to blood-testis barrier (BTB) disruption and a concurrent increase in the number of peritubular macrophages, unlike exposure to the specific spermatocyte germ cell toxicant Methoxyacetic acid (MAA) (Fang et al., 2024a). This underlines the necessity of BTB disruption for potential leak out of luminal apoptotic germ cell contents to reach PTMCs, promoting MCP-1 production. This recent finding aligns with our earlier observation that a single high dose of MEHP resulted in a six-fold increase in immune cells within the rat testis, predominantly peritubular macrophages (Gillette et al., 2021). In this study, we were trying to understand whether the sequence of events triggered by MEHP exposure—specifically, 1) mass apoptosis of germ cells, 2) disruption of the blood-testis barrier (BTB), and 3) the leakage of damaged spermatocyte/spermatogonia particles reaching PTMCs—is essential for MCP-1 production. To explore this, we tested the null hypothesis that these prerequisite events do not play a role in MEHP-induced MCP-1 secretion by PTMCs.

For the first time, we report that PTMCs produce MCP-1 in vitro when directly exposed to MEHP, even in the absence of prerequisite events, and in vivo in their presence. Our proteomic analysis and quantitative PCR results further support this novel discovery, which reveals that MEHP exposure stimulates MCP-1 production in PTMCs via the IL-1 signal transduction pathway. The new understanding of the regulation of MCP-1 production by PTMCs is particularly noteworthy, as it underscores their crucial role in modulating immune responses within the SSC niche. This finding advances our knowledge of PTMCs’ function and opens new avenues for research into immune regulation and potential therapeutic interventions targeting the SSC niche.

## 2. Material and methods

### 2.1. Chemicals, reagents, and antibodies

MEHP (purity 97.3 %) was purchased from Wako Chemicals (Richmond, Virginia). Iodine (Cas# 7553–56–2), collagenase from *Clostridium histolyticum* (Cat # 9001–12–1), DNase I from bovine pancreas (Cat# 11284932001), hyaluronidase from bovine testis (SKU H 3506), and PureProteome albumin magnetic bead suspension (Cat # LSKMAGL10) were purchased from Millipore Sigma. Dulbecco’s phosphate-buffered saline (PBS) without Ca^2+^ and Mg^2+^ (Cat # 14190144), Gibco glucose solution (Cat# A 2494001), 100X penicillin/streptomycin-glutamine (Cat# 10378016), trypsin (1:250) powder (Cat # 36978), heat-inactivated fetal bovine serum (FBS), RPMI-1640, paraformaldehyde (PFA), actin (smooth muscle) (Ref. MS – 113 – PO), MCP-1 (CCL2) antibody (PA1–22488), radioimmunoprecipitation assay (RIPA) lysis and extraction buffer (Cat# 89900), and Halt protease and phosphatase inhibitor cocktail (Cat# 78440) were purchased from Thermo Fisher (Waltham, Massachusetts). Alexa Fluor 488 goat anti-mouse secondary antibody (Cat # A11001), GAPDH polyclonal antibody (Cat # PA1–987), NLRP3 polyclonal antibody (Cat# PA5–79740), and IL-1R1 polyclonal antibody (Cat# PA5–96408) were purchased from Invitrogen (Waltham, Massachusetts).

### 2.2. Animals

All experimental animal work received approval from the Institutional Animal Care and Use Committee (IACUC) at the University of Texas at Austin, and the animals were maintained according to the standards detailed in the *Guide for the Care and Use of Laboratory Animals*. The University of Texas at Austin is an AAALAC International accredited institution; as a result, the ARRIVE guidelines were followed in preparing this manuscript. Fisher CD 344 male rats (Fisher) at postnatal day (PND) 21 were purchased from Charles River (Wilmington, Massachusetts) and arrived at PND 25. Upon arrival, the animals were housed in temperature-controlled conditions with a 12:12 light-to-dark cycle and provided with *ad libitum* food and water. The animals were randomly grouped and allowed to acclimatize for 3 days before the experiments began.

### 2.3. MEHP exposure

Five rats were administered MEHP or corn oil on postnatal day 28 via oral route. MEHP was diluted in corn oil and delivered through oral gavage at a dose of 700 mg/kg body weight, or an equivalent volume of corn oil was given to the control group of rats. After the designated exposure period, the rats were euthanized using CO_2_ asphyxiation, followed by cervical dislocation and thoracic puncture, and the testes were quickly removed and frozen. The *in vitro* exposure concentration (Hong et al., 2021) and *in vivo* exposure dose (Murphy et al., 2014) of MEHP were determined based on our previous study, which indicated that a similar in vivo dose could elicit a significant increase in MCP-1 and leukocyte infiltration in the rat testis.

### 2.4. Isolation, culture, and treatment of primary PTMC isolates

Ten PND 21 rats were used to isolate PTMCs. Male pups at PND19 were sent to us along with their dams. The animals were euthanized using CO_2_ asphyxiation, followed by cervical dislocation and thoracic puncture, after which the testes were removed. Primary cultures of PTMCs were isolated from rat testis following the established method (Bhushan et al., 2016). The testes were taken from the animals and disinfected with iodine in ethanol. Seminiferous tubules were extracted and digested in a Trypsin-DNase I solution. Once the tubules were fully dispersed in the solution, a trypsin inhibitor solution was added to stop further digestion, and the solution was washed several times to remove any residual trypsin. The next step involved digestion in a Collagenase-Hyaluronidase-DNase I solution for 10 minutes in an oscillating water bath. After confirming the detachment of PTMCs from the seminiferous tubules through light microscopy, the suspension was allowed to stand for 10 minutes to facilitate the sedimentation of the remaining tubular portions. At this stage, the PTMCs remained in suspension; thus, the supernatant was collected while the sediment was discarded. The isolated cells were cultured in RPMI 1640 supplemented with 10 % fetal bovine serum and 1 % streptomycin/penicillin. The primary culture was first sub-cultured on the third day of isolation, then again on the seventh day, and seeded into 120 mm circular discs for protein isolation and T75 flasks for RNA isolation. Meanwhile, cells were grown on slides to assess purity.

**Fig. 1.**
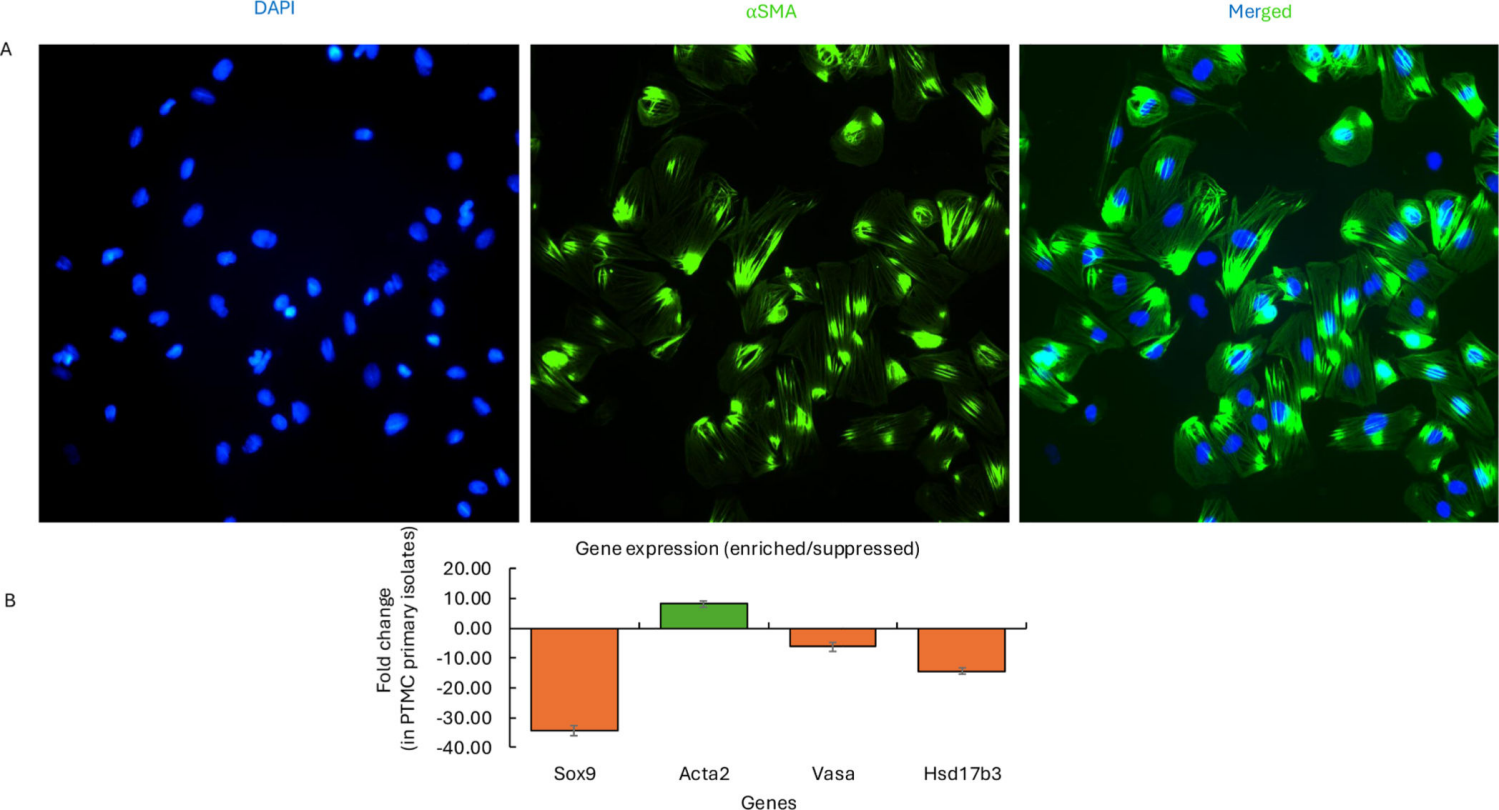
Assessment of the purity of PTMC primary isolates from rat testis. **A.** PTMC marker smooth muscle actin-alpha (⍺SMA) was used to evaluate the purity of primary isolates. **B.** Signature gene expression analysis in PTMC primary isolates: Sertoli cells, Sox9; germ cells, Vasa; Leydig cells, Hsd17b3; and PTMCs, Acta2; using qRT-PCR. Please refer to the supplementary information for details on the calculation.

The purity of the cultures was evaluated using the alpha-smooth muscle actin (α-SMA) antibody marker and marker gene expression analysis via qRT-PCR (Fig. 1A & B). RPMI-1640 supplemented with 10 % FBS and 1 % antibiotics was used as the media in an incubator with 5 % CO_2_ at 37 °C. After the second passage, the cells were treated with 100 μM and 200 μM MEHP for 12 or 48 hours before collecting protein and mRNA.

### 2.5. Immunocytofluorescence

PTMCs cultured on slides were washed and fixed in 4 % PFA for 10 minutes at room temperature (RT). They were then incubated in 5 % Bovine Serum Albumin (BSA) at RT for 1 hour and exposed to the primary antibody α-SMA at a dilution of 1:100 overnight at 4°°C. The next day, the slides were washed with PBS-0.1 % Tween 20 and treated with a secondary antibody, AF 488 goat anti-mouse, at a dilution of 1:500 at RT for 1 hour in darkness. Finally, after several washes with PBS-0.1 % Tween 20, mounting was performed using 4^’^, 6-diamidino-2-phenylindole (DAPI).

### 2.6. Immunofluorescent staining of testis cross-sections

Paraffin-embedded tissue blocks were prepared following necessary tissue pre-processing. Five-micrometer sections were sliced from the paraffin-embedded tissue blocks using a microtome HM355S (Thermo Scientific) and mounted on glass slides. After dewaxing and rehydration, the sections were immersed in citrate buffer solution (10 mM, pH 6) and microwaved to retrieve the antigen. Incubation in 3 % hydrogen peroxide solution for 15 minutes effectively blocked endogenous peroxide activity. The sections were then incubated in goat-blocking serum, followed by overnight exposure to the primary antibody, MCP-1 Antibody (PA1–22488, Thermo Fisher), at a dilution of 1:500, at 4°C. The secondary antibody, Alexa Fluor 568 goat anti-rabbit, at a dilution of 1:500, was applied for one hour at room temperature in a dark environment the following day. Counterstaining was performed with DAPI, and the sections were cover-slipped for microscopic observation.

### 2.7. Isolation of interstitial fluid (IF)

The basal compartment milieu of the testis is fully represented by IF. To identify the cytokines and chemokines involved in MEHP-induced testicular injury, we treated five PND 28 rats with 700 mg/kg body weight of MEHP dissolved in corn oil, while five additional rats received corn oil only. After 6 hours of exposure, we isolated the interstitial fluid (IF) from the testis. The IF isolation was adapted from a previous protocol (Mittenbühler et al., 2023). Briefly, after extracting the testis, we carefully opened the tunica layer and placed it in a centrifuge tube containing a 20-micron nylon mesh (Millipore Sigma, 2004700), followed by centrifugation. The IF collected at the bottom of the tube was stored at −80°C until analysis.

### 2.8. Protein sample (isolation, quantification, and enrichment)

Protein lysates were collected using RIPA buffer supplemented with a protease inhibitor cocktail. The samples for the proteomics experiment were immunodepleted with Albumin Magnetic beads. Prior to immunodepletion, protein quantification was performed using the Pierce BCA Protein Assay Kit (Thermo Scientific, Cat# 23227). A total of 250 μg of protein from cell lysates was diluted in PBS to a final volume of 100 μL. The diluted protein lysate was combined with an equilibrated PureProteome albumin magnetic bead suspension (750 μL) and incubated at room temperature for 1 hour with end-over-end mixing. The tubes were then placed on a magnetic stand, allowing the beads to attach to the magnet. While the beads remained attached to the magnet, the supernatant (depleted protein sample) was collected in a separate tube. The beads were washed three times with 500 μL PBS and combined in the same collection tube to optimize recovery. The depleted samples pooled with wash fractions resulted in a dilute solution, which was subsequently concentrated using an Amicon Ultra-4 Centrifugal Filter Unit with a 3 K MWCO. Concentrated samples were then quantified again with the Pierce BCA Protein Assay Kit (Thermo Scientific, Cat# 23227), and a total of 20 μg of each sample in 100 μL was submitted for proteomic analysis.

### 2.9. RNA isolation, quantification, and quality assessment

RNA samples from each group were isolated using the PureLink RNA Mini Kit (Invitrogen, Cat# 12183018 A) according to the manufacturer’s instructions. Initial quantification was performed using a Nanodrop 2000c spectrophotometer. Next, following the manufacturer’s protocol, a QC check was conducted using the Agilent RNA 6000 Nano Kit (reorder number 5067–1511). The ladder was prepared upon arrival and stored at −70°C. Once the electrodes were decontaminated, the dye was mixed with the ready-to-use gel and kept in the dark until needed. A fully thawed gel-dye mixture, along with the RNA 6000 Nano Marker, was loaded into the well, followed by samples from the remaining wells. The results were interpreted according to the manufacturer’s guidelines.

### 2.10. Multiplex assay

The multiplex assay utilized a MILLIPLEX MAP Rat Cytokine/Chemokine Magnetic Panel (Millipore, Cat# RECYTMAG-65K). The experiment was conducted according to the manufacturer’s instructions. Briefly, 200 μL of assay buffer was added per well and sealed to mix on a plate shaker for 10 minutes at room temperature. Twenty-five microliters (25 μL) of standard or control were added to the appropriate wells, along with 25 μL of assay buffer to the sample and background wells, followed by the addition of matrix solution (25 μL) to the background, standards, and control wells. At this stage, 25 μL of the sample was placed into the sample wells, and the plate was incubated for 2 hours at room temperature. At the end of the incubation period, the plate was washed twice with wash buffer after carefully removing the contents from the wells. The detection antibodies were added (25 μL per well) and sealed for incubation for 1 hour on a plate shaker at room temperature. After the second incubation, 25 μL Streptavidin-Phycoerythrin was added to each well, each containing 25 μL of antibodies. The third or final incubation of the plate was performed on a plate shaker for 30 minutes at room temperature. The contents in the wells were carefully removed and washed with 200 μL wash buffer twice. The sheath fluid per well was added, and beads were resuspended using a plate shaker for 5 minutes. Finally, the plate was analyzed using a MAGPIX instrument with xPONENT software. Although we considered three experimental replicates in the multiplex assay design, we did not conduct a statistical test to determine the significance level. The reason was that interstitial fluid (IF) from individual animals’ testes was insufficient for the experiment, so we had to pool IF from the control group (five) and IF from the treatment group (five) into two tubes. A pooled sample enables researchers to detect analyte signals more efficiently by minimizing noise from individual differences. Moreover, pooled samples provide a distinct advantage when the overall trend of the analytes is prioritized over individual differences.

### 2.11. qRT-PCR analysis of selected IL-1 signaling pathway genes

Total RNA was isolated from PTMC primary isolates using the PureLink RNA Mini Kit (Invitrogen). Gene expression was evaluated using a two-step protocol. First, the cDNA pool was prepared through reverse transcription of RNA. In the second step, quantitative real-time polymerase chain reaction (qRT-PCR) with SYBR Green was performed using a kit from Bio-Rad. The CFX96 RT-PCR system (Bio-Rad) was programmed to 95°C for 3 minutes, followed by 39 cycles, each consisting of 95°C for 30 seconds and 60°C for 30 seconds, with a final step of 72°C for 1 minute for PCR amplification. The primers used in this study are listed in Supplementary Table 1. The specificity of the PCR product was evaluated through melt curve analysis, which was set between 60°C and 95°C with increments of 0.5°C. The cycle threshold (Ct) values obtained for the genes were first normalized with a housekeeping gene (GAPDH) and subsequently with a control sample. The fold change was calculated using the delta-delta Ct (-ΔΔCt) method (Schmittgen and Livak, 2008). The housekeeping gene (GAPDH) was selected based on the results of reference gene selection tools (Vandesompele et al., 2002) utilizing CFX Maestro software, further supported by a previous study (Liu et al., 2020). Briefly, the mRNA expression levels of five potential reference genes (GAPDH, β-actin, 18S, RPS16, and RPL19) were assessed using qRT-PCR, and the data were utilized to calculate M values for evaluating the stability of gene expression. GAPDH exhibited a smaller M value, indicating more stable gene expression across sample types. Three experimental replicates in each group were included in this experiment.

### 2.12. Proteomics

Two samples from each group were digested with trypsin, desalted, and subsequently analyzed using the Dionex LC and Orbitrap Fusion 2 for LC-MS/MS, with a runtime of 60 minutes. The resulting MS/MS peptide spectra were compared against a protein sequence database to identify parent protein molecules, and their quantification was achieved by analyzing the peak intensities of relevant peptides with the software. Raw data files were examined using Proteome Discoverer version 2.5.0.400 and Scaffold version 5.3.0. The protein lysates used for the proteomics analysis consist of a pooled sample. While pooled samples efficiently utilize resources, the integrity of the data remains preserved. Pooled samples allow researchers to detect analyte signals more effectively by reducing noise from individual differences. Since we aimed to understand the overall trends of the analytes (IL-1 signaling pathway proteins), using pooled samples best met our needs even without applying statistical tests. The University of Texas at Austin Center for Biomedical Research Support Biological Mass Spectrometry Facility provided protein identification (RRID: SCR_021728).

### 2.13. RNA sequencing library preparation

Four RNA samples from each group were first quantified using Qubit analysis, followed by bioanalyzer assessment with the DNA: First Available Chip. Subsequently, the RNA samples underwent ribosomal depletion, and RNA-Seq library preparation was conducted with NovaSeq SP PE150 (per read), targeting 50 million 150 bp read pairs per library. All sequencing work was performed at the University of Texas at Austin Genomic Sequencing and Analysis Facility.

#### 2.13.1. RNA seq data analysis

Raw reads were mapped to the rat reference transcriptome using Kallisto version 0.46.2. The mapping report was generated using MultiQC and imported into the R environment with the TxImport package. The data were then normalized using the Trimmed Mean of M-values (TMM) method, and upregulated or downregulated genes were determined through linear modeling and Bayesian statistics. Differentially expressed genes were identified by applying precision weights to individual genes based on their mean-variance relationship. Pearson correlation was used to cluster differentially expressed genes presented on a heatmap, with data arranged by Z-score for each row. Gene Ontology (GO) enrichment, followed by Gene Set Enrichment Analysis (GSEA), was performed to identify enriched gene sets.

### 2.14. Statistical analysis

P values were calculated using a one-tailed Student’s *t*-test with unequal variance. The displayed data represent means ± SEM. The corresponding figure legends indicate the statistical tests used to determine the P values.

## 3. Results

### 3.1. Elevated level of cytokines and chemokines in the testis IF

**Fig. 2.**
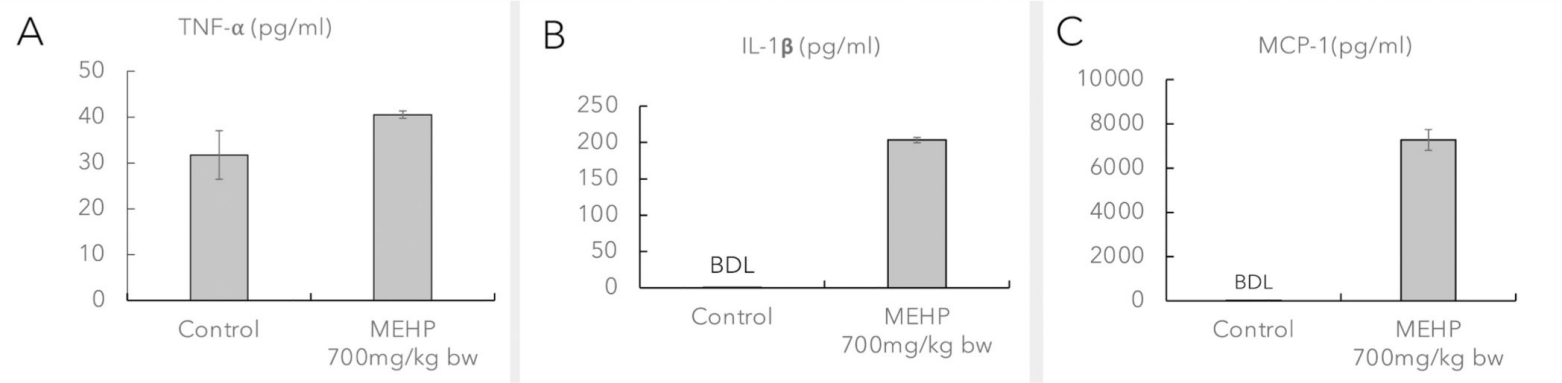
Changes in cytokines and a chemokine in the interstitial fluid from rat testis from control animals and those exposed to MEHP (700 mg/kg-bw) **A.** TNF-⍺ **B.** IL-1β **C.** MCP-1. BDL-below the detection level.

The multiplex assay detected chemokines and cytokines in the interstitial fluid, particularly IL-1β, TNF-α, and MCP-1. Our results indicated that IL-1β changed drastically in the treatment group (MEHP 700 mg/kg bw), while in the control group, it remained below the detection level. Additionally, the TNF-α level in the MEHP treatment group was observed to be 7 pg/ml higher than that in the control group. The chemokine MCP-1, which is responsible for macrophage infiltration, significantly increased following MEHP exposure (Fig. 2 A, B, & C).

### 3.2. RNA sequencing

#### 3.2.1. Greater Differentially Expressed Genes (DEGs) were observed in PTMC primary isolates from rat testis exposed to MEHP 200μM for 48 h

**Fig. 3.**
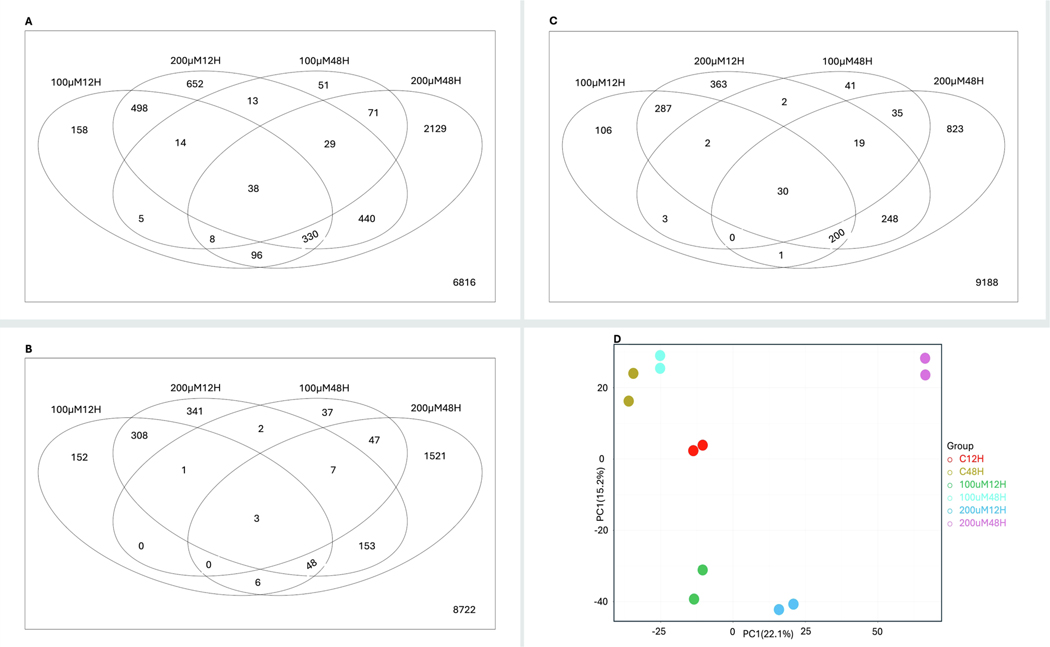
Commonalities and differences in the differential gene expression in each treatment group. **A.** Total DEGs (including up and down-regulated genes). **B.** Upregulated DEGs. **C.** Down-regulated DEGs. The individual circle in the Venn diagram represents a treatment group, and the number outside the circle indicates the number of genes that do not belong to any sets. DEGs were selected using p-value = 0.05 and log base2 fold change equal to or greater than 0.5. **D.** Principal component analysis (PCA) to visualize the variation among six samples along the PC1 and PC2, in which PC1 was 22.1 % and PC2 was 15.2 %. Normalized (reads per kilobases of transcript per 1 million mapped reads) and log-transformed count data were used for PCA analysis.

Mild to moderate changes in gene expression were observed with 100 μM MEHP exposure at both 12 and 48 hours; however, a clear distinction was noted between the control and 200 μμM MEHP exposures at both time points. A total of 4532 differentially expressed genes (DEGs) were identified across all groups, with a greater number of DEGs detected in the 200 μM MEHP exposure group at 48 hours compared to the corresponding 12-hour exposure group (Fig. 3A). Notably, 863 genes were upregulated (Fig. 3B), while 1151 genes were significantly downregulated (Fig. 3C) in the 12-hour 200 μM MEHP exposure group. In contrast, 1785 genes were upregulated (Figs. 3B), and 1356 genes were significantly downregulated in the 48-hour 200 μM MEHP group (Fig. 3C).

**Fig. 4.**
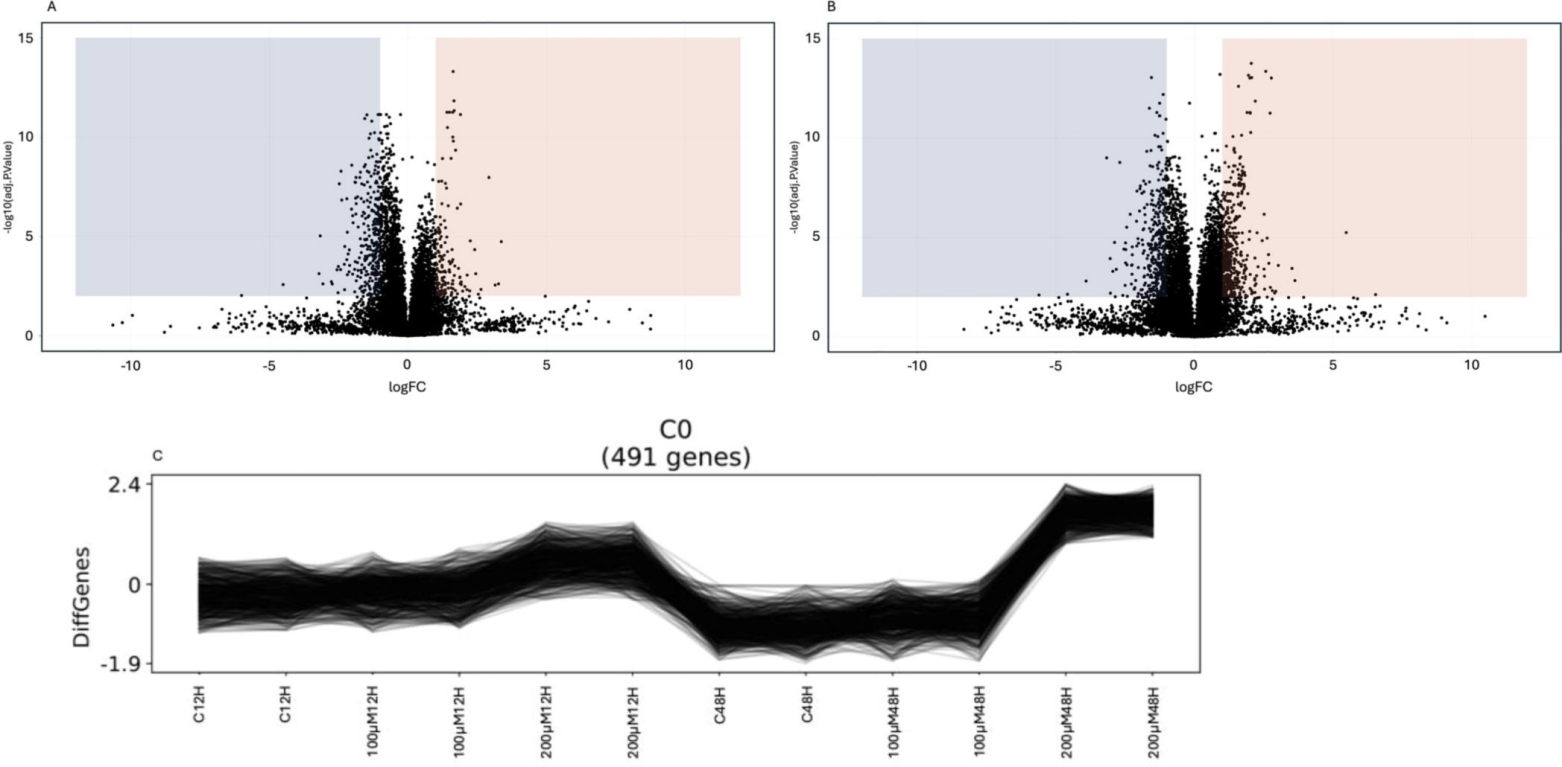
Identification and visualization of DEGs. Volcano plot displaying log_2_ fold change versus –log_10_ P value to demonstrate statistically significant DEGs within the exposure group. **A.** MEHP 12 h exposure **B.** MEHP 48 h exposure. **C.** Clustering analysis using (*Clust*) hybrid ‘Bi-CoPaM’ approach. *Clust* can extract clusters of genes consistently co-expressed from different gene expression datasets with various conditions and replications. After filtering, 4532 differentially expressed genes *Clust* function was utilized to produce clusters. Four hundred ninety-one genes were co-expressed in all 12 h and 48 h groups and at distinctly higher levels with 200 μM MEHP exposure for 48 hours. DEGs were selected using p-value = 0.05 and log base2 fold change equal to 0.5.

The principal component analysis (PCA) identified three sources of variation. The first component (PC1) explained 22.1 % of the variation, while the second component (PC2) accounted for 15.2 % (Fig. 3D), and the third component (PC3) contributed approximately 11 % of the variation (data not shown). The separation of sample groups, including control, MEHP 100μM, and MEHP 200μM, at both exposure time points indicated that PC1 represented the MEHP exposure concentration, whereas PC2 represented the duration of exposure. Although the batch effect was observable, the higher concentration and longer duration of MEHP exposure led to more noticeable changes in the transcriptome (Fig. 3D). Furthermore, the volcano plot displayed a significantly greater number of genes upregulated in the 48-hour exposure group compared to the 12-hour MEHP exposure group (Fig. 4A&B).

#### 3.2.2. At both exposure time points, exposure to 100 μM MEHP caused less apparent changes in the PTMC transcriptome

**Fig. 5.**
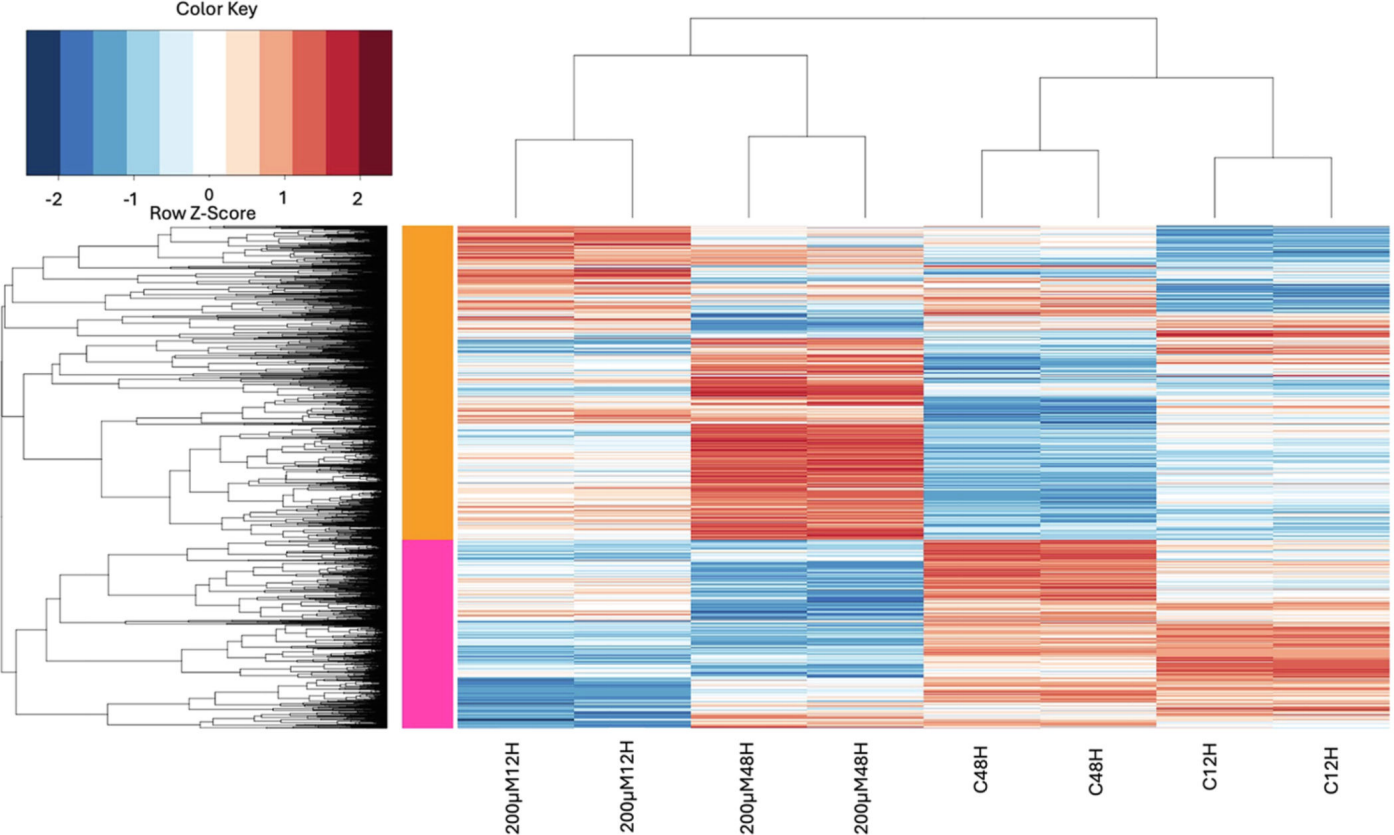
Heat map demonstrating modules of upregulated and downregulated genes upon exposure to 200 μM MEHP for 12 and 48 hours. The Z-score bar indicates a relative expression + /− standard deviation from the mean. The False discovery rate (FDR) is less than 0.05. DEGs were selected using p-value = 0.05 and log base2 fold change equal to 0.5.

As shown in the DEGs, more significant changes in gene expression were observed in the 48 h MEHP exposure group compared to the 12 h MEHP exposure group. Results from the PCA plot, volcano plot, clustering analysis (Fig. 4C), and heatmaps clearly indicate that 100 μM MEHP exposure causes less noticeable changes in gene expression at both 12 h and 48 h of exposure (Supplementary Fig. 1A). In contrast to the 12 h 200 μM MEHP exposure group, the cluster of genes that were upregulated in the 48 h 200 μM MEHP exposure group was distinctly different (Supplementary Fig. 1B). However, the downregulation of genes in these groups varied slightly (Supplementary Fig. 1C). Interestingly, a cluster of genes that were notably downregulated in the 12 h 200 μM MEHP exposure remained less affected in the 48 h 200 μM MEHP exposure. Furthermore, a cluster of genes that were less affected in the 12 h 200 μM MEHP exposure was found to be further downregulated in the 48 h 200 μM MEHP exposure, indicating that various gene clusters respond to toxicants at different time points (Fig. 5).

#### 3.2.3. GO enrichment, GSEA, and targeted pathway analyses

##### 3.2.3.1. Gene Ontology (GO) analysis of the top differentially expressed genes (DEGs) indicated that numerous pathways related to the immune and inflammatory response were significantly enriched.

The pathways associated with Molecular Functions (MF), Cellular Compartments (CC), and Biological Processes (BP) were specifically examined, alongside relevant pathways from the Kyoto Encyclopedia of Genes and Genomes (KEGG), the REACTOME database (which is free, open-source, curated, and peer-reviewed), Transcription Factors (TF), and the Wiki Pathways (WP) database. A subset of the most relevant Gene Ontologies (GOs) included the inflammatory response, immune response, regulation of inflammatory response, positive regulation of Nuclear Factor Kappa B (NF-kB) transcription factor activity, innate immune response, protein binding, positive regulation of cytokine production involved in immune response, cytokine production involved in immune response, regulation of cytokine production, immune system process, activation of immune response, positive regulation of the production of molecular mediators of immune response, immune response activating signaling pathway, and positive regulation of immune response (Supplementary Fig. 2).

Pathways found in the KEGG and REACTOME databases were also significantly enriched. A subgroup of these pathways associated with the immune response includes the NOD-like receptor signaling pathway, MAPK targets/Nuclear events mediated by MAP kinases, ERK/MAPK targets, Interleukin-17 signaling, Toll-like Receptor 4 (TLR4) cascade, MyD88:MAL (Myeloid differentiation primary response 88) and MAL (also known as TIRAP, Toll-interleukin 1 receptor domain-containing adaptor protein) cascade initiated on the plasma membrane, TNF signaling, and Nucleotide-binding domain, leucine rich repeat containing receptor (NLR) signaling pathways (Supplementary Fig. 2).

Likewise, the transcription factor (TF)- associated Gene Ontology (GO) enrichment analysis results indicated significant enrichment of TFs known to regulate genes involved in immune response, such as NF-kB, C/EBPdelta, ATF-1, ATF2:cJun, CREB, and AP. Additionally, the WikiPathways (WP)- associated GO enrichment analysis revealed significant enrichment of the p38 MAPK signaling pathway and the TNF-alpha NF-kB signaling pathways (Supplementary Fig. 2).

##### 3.2.3.2. Gene set enrichment analysis (GSEA) indicated that the Interleukin-1 signaling pathway was significantly enriched after 48 hours of 200 μM MEHP exposure but not after 12 hours.

Gene set enrichment analysis (GSEA) revealed that 531 and 542 pathways were enriched following 12-hour and 48-hour exposures to 200 μM MEHP, respectively. In the 12-hour exposure group, 63 pathways were significantly upregulated, with normalized enrichment scores (NES) ranging from 2.25 to 1.36, while 468 pathways were significantly downregulated (NES range −2.44 to −1.37). Similarly, in the 48-hour exposure group with 200 μM MEHP, 180 pathways were significantly enriched (NES range 3.67–1.34), and 362 pathways were significantly downregulated (NES range −2.41 to −1.30). It was evident that more pathways or gene sets were upregulated in the 48-hour exposure to 200 μM MEHP compared to the 12-hour exposure at the same concentration (data not shown).

**Fig. 6.**
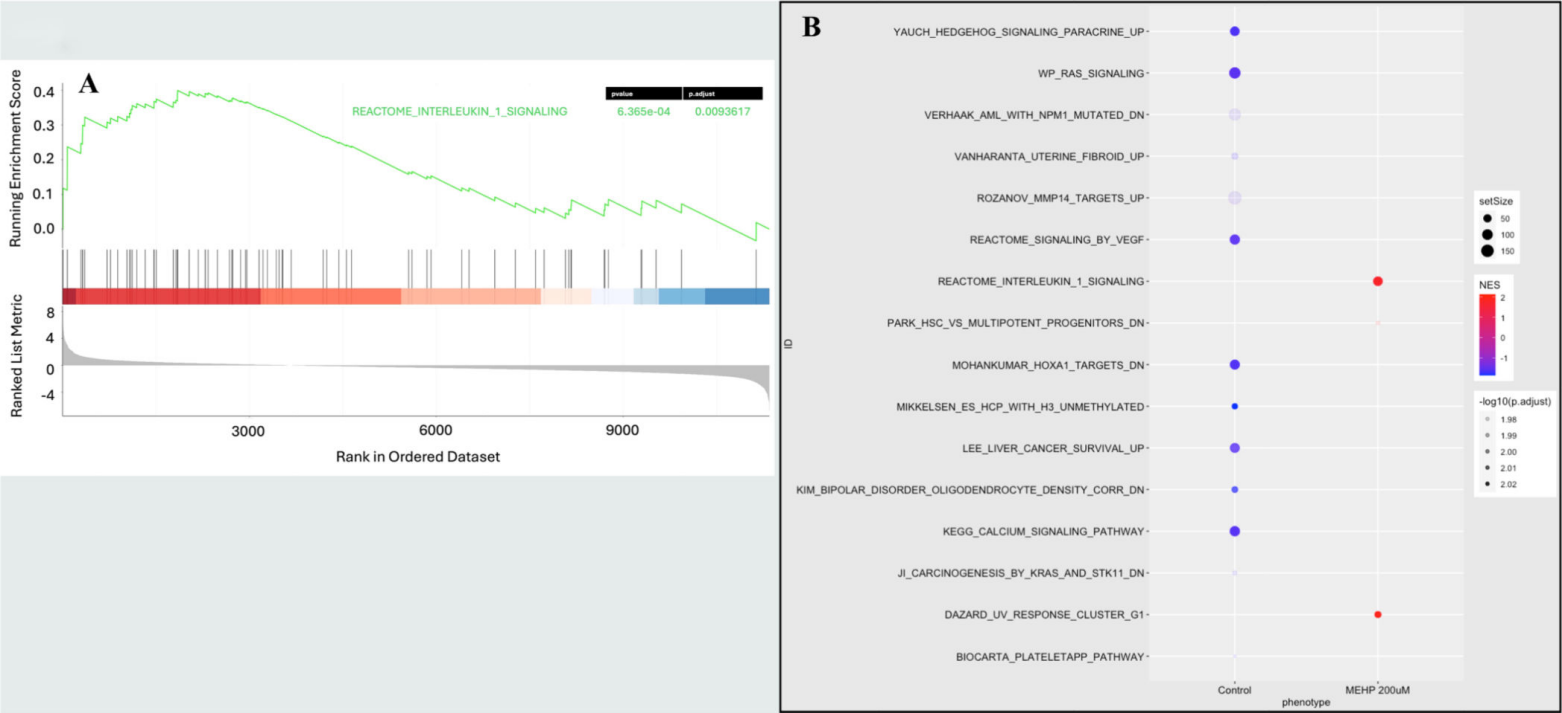
Functional enrichment analysis using Gene Set Enrichment Analysis (GSEA) to identify a specific pathway enrichment status in the group MEHP 200 μM exposed for 48 hours. **A.** Reactome IL-1 signaling pathway enrichment. **B.** Bubble plot demonstrating a significant enrichment of IL-1 signaling pathway.

**Fig. 7.**
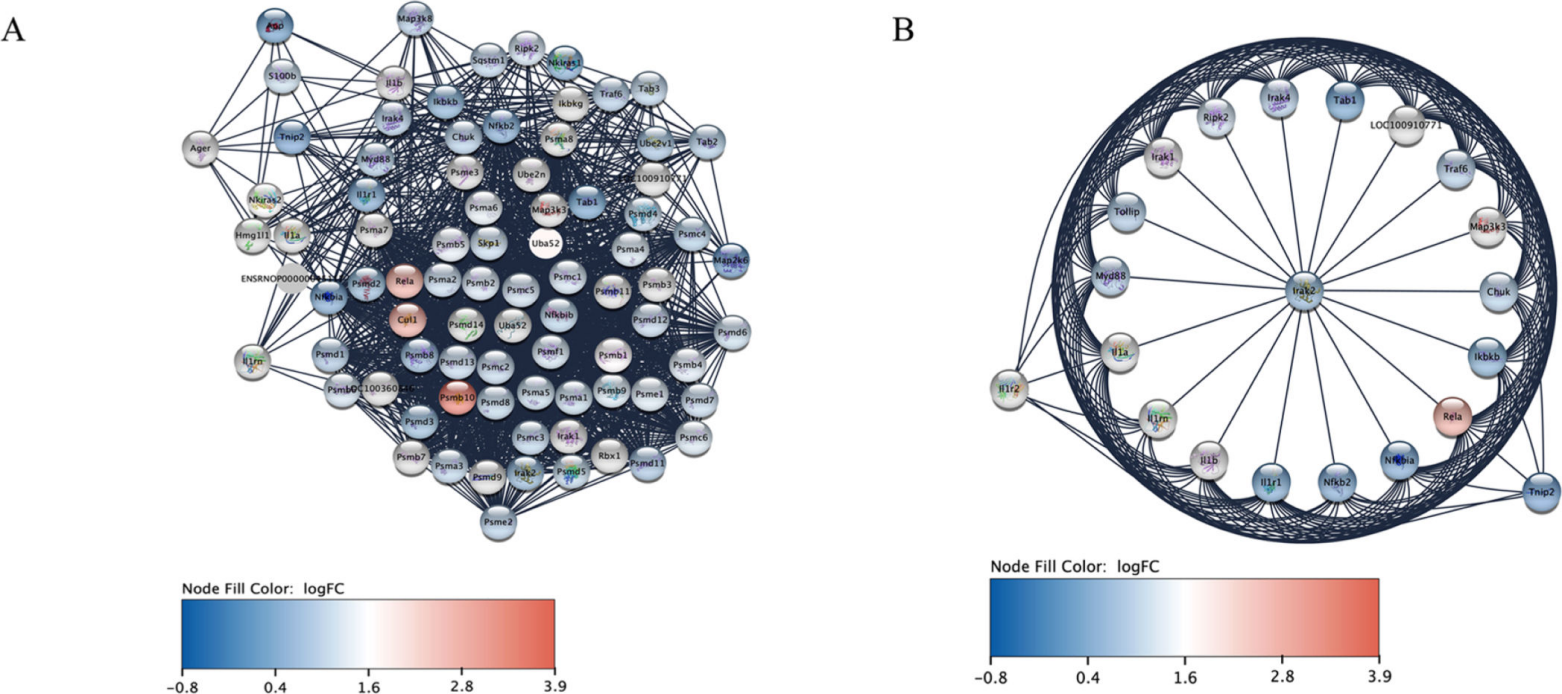
Visualization of Rat IL-1 signaling pathway protein network. **A.** The IL-1 signaling pathway protein network was supplemented with transcriptomics data (200 μM MEHP exposure for 48 hours). **B.** Protein-protein interactions (PPIs) network of Rela protein supplemented with transcriptomics data (200 μM MEHP exposure for 48 hours). Images were produced by Cytoscape software. Proteins or associated genes are represented by nodes and interactions by edges. The scale bar represents log_2_ fold change.

Among 63 statistically significant enriched pathways identified after 12-hour exposure to 200 μM MEHP, many were associated with DNA damage repair (10), cellular respiration, cellular stress response, epigenetic modifications, cell cycle regulation, and translation. Interestingly, gene sets related to the inflammatory response showed statistically significant negative enrichment scores, while gene sets associated with oxidative phosphorylation and cellular response to starvation were significantly upregulated. Following 48-hour exposure to 200 μM MEHP, in addition to the pathways upregulated in the 12-hour exposure group, many other pathways became significantly enriched. Notably, pathways linked to immune response were identified, including the Notch4 signaling pathway (NES 2.40) (not shown), the FCERI-mediated NF-kB activation pathway (NES 2.48) (not shown), the Dectin-1-mediated non-canonical NF-kB signaling pathway (NES 2.33), the B Cell receptor signaling pathway (NES 2.33), T Cell receptor signaling (NES 1.84), CLEC7A Dectin-1 signaling (NES 1.79) (Supplementary Fig. 3), and the Interleukin-1 signaling pathway (NES 1.75) (Fig. 6A & B, Fig. 7 A & B).

### 3.3. Results from proteomics showed an increase in proteins in the Interleukin-1 signaling pathway

Our proteomics results indicated that proteasomes, ubiquitin, and other protein groups associated with IL-1 signaling were upregulated in PTMC isolates treated with MEHP (Table 2). Subunits of the proteasome, including proteasome subunit alpha (Psma types 3–6), proteasome subunit beta (Psmb types 4 and 10), 26S proteasome regulatory subunits (Psmc types 1–6), non-ATPase regulatory subunits of the 26S proteasome (Psmd types 1, 5, 8, 11, and 13), along with proteasome activator subunit 4 (Psme4), ubiquitin-60S ribosomal protein L40 (Uba52), Cullin 1 (Cul1), mitogen-activated protein kinase 1 (Mapk1), extracellular signal-regulated kinase 2 (Erk2), and Fos-related antigen 2 were also upregulated in the MEHP-treated group (Table 2).

### 3.4. Gene expression analysis using qRT-PCR further supported the results of the RNA-seq analysis

**Fig. 8.**
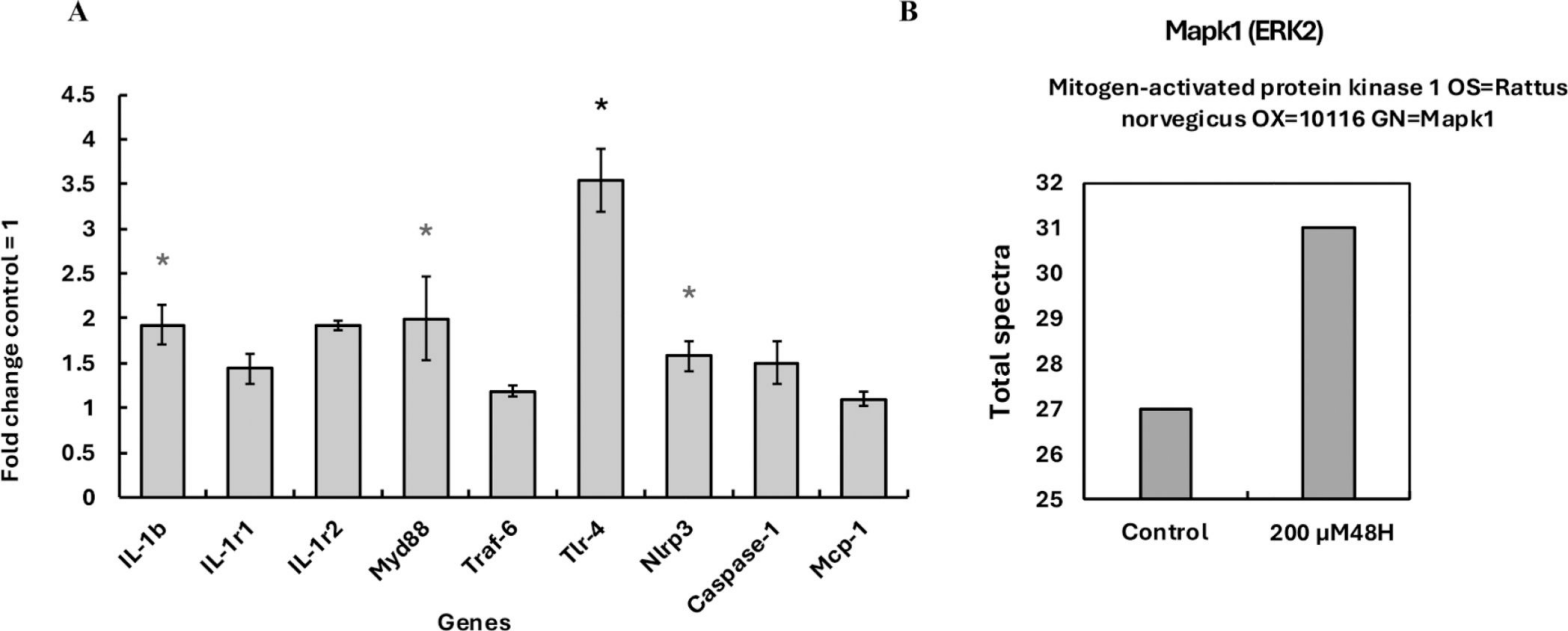
Cross-validation of transcriptomics results. **A.** qRT-PCR analysis of IL-1 Signaling pathway gene expression in PTMC primary isolates exposed to 200 μM MEHP for 48 hours. **B.** Mapk1/ERK2 protein expression analysis through proteomics in PTMC primary isolates exposed to 200 μM MEHP for 48 hours. Scaffold alias v5 software was used for the identification and quantification of protein. Student’s *t*-test, with unequal variance, was performed for the statistical significance, *p-value < 0.05.

As we observed, the IL-1 signaling pathway was enriched after 48 hours of exposure to 200 μM MEHP in PTMC primary isolates. Nine genes from the IL-1 signaling pathway were selected for qRT-PCR gene expression analysis. The genes IL-1β (1.93 ± 0.22 fold), MyD88 (2.0 ± 0.46 fold), TLR-4 (3.54 ± 0.34 fold), and Nlrp3 (1.58 ± 1 fold) were found to be statistically significantly upregulated, while IL-1r1 (1.44 ± 0.16 fold), IL-1r2 (1.91 ± 0.5 fold), Traf6 (1.19 ± 0.05 fold), Caspase 1 (1.50 ± 0.23 fold), and the chemokine MCP-1 (1.10 ± 0.06 fold) showed a distinct upregulation. Likewise, the RNA-seq data revealed several fold-upregulations of Nlrp3, Map3k7/Tak1, and Mapkapk2/Mk2 compared to the control group (Supplementary Figure 4B). Notably, the Rela transcript was found statistically significantly differentially expressed in the 48-hour 200 μM MEHP exposure group. Overall, our qRT-PCR results aligned with our RNA-seq results, supporting the idea that the RNA-seq analysis was accurate (Fig. 8A).

### 3.5. A crucial protein in the IL-1 signaling pathway

Through proteomics analysis, the key protein in the IL-1 signaling pathway, ERK2, was shown to increase in the protein lysates of the 48-hour 200 μM MEHP exposure group (Fig. 8B). This finding supported the results of transcriptomics and qRT-PCR.

### 3.6. Demonstration of MCP-1 expression by PTMCs through immunofluorescence staining of testis sections – in vivo

**Fig. 9.**
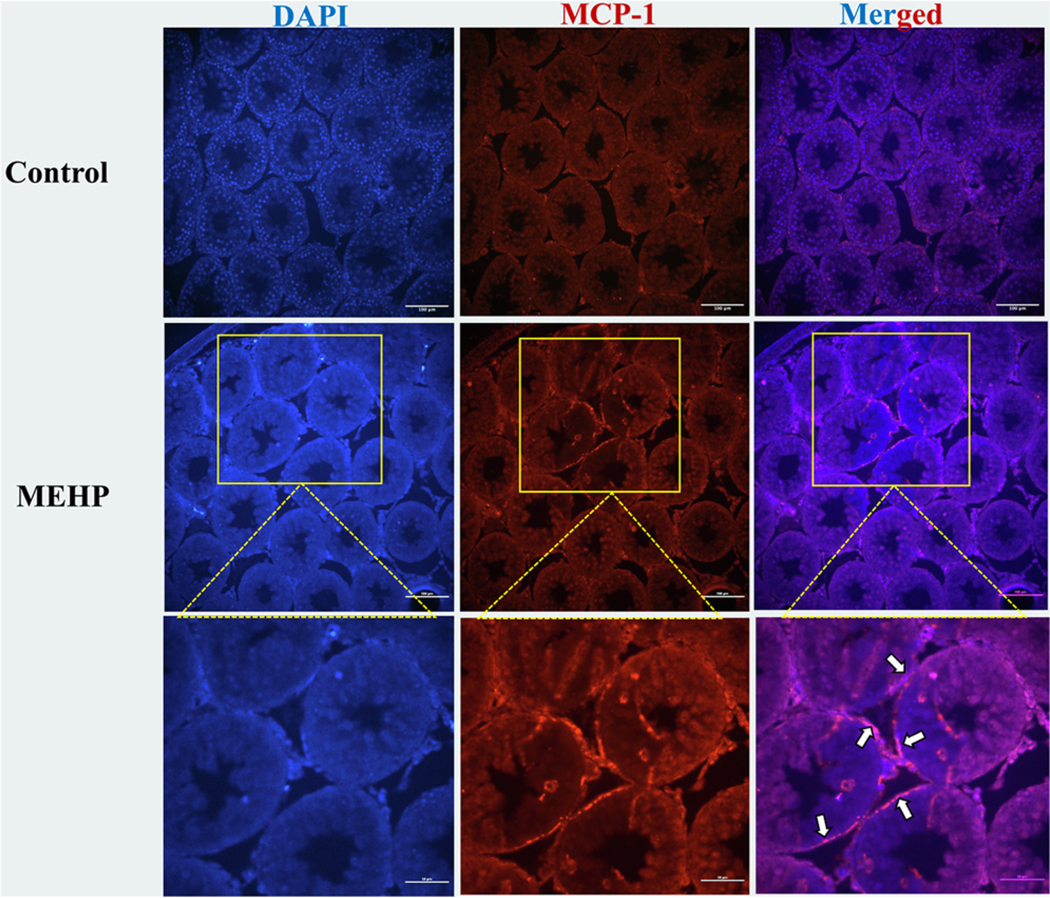
MCP-1 secretion by PTMCs. An immunofluorescence study was performed on testis sections from rats exposed to MEHP (700 mg/kg bw) for 48 hours. White arrows indicate MCP-1 secretion by PTMCs surrounding seminiferous tubules (scale bar = 100 μm & 50 μm).

MCP-1 secretion by PTMCs was assessed in testis sections from 28-day-old Fisher CD344 rats exposed to MEHP. PTMCs are present as a single layer of cells situated just above the basement membrane surrounding the seminiferous tubules (ST). A distinct level of MCP-1 signals was observed in the peripheral region of the seminiferous tubules where PTMCs are located, as illustrated in Fig. 9. Very few MCP-1 signals were detected in the control group compared to the treatment group, as expected since MCP-1 is also secreted by Leydig cells and immune cells. This indicates that MEHP exposure stimulated MCP-1 production by PTMCs.

## 4. Discussion

In this study, we demonstrated that isolated PTMCs produce MCP-1 upon direct exposure to MEHP and elucidated the signal transduction pathway involved in this process. Also, we demonstrated that MEHP exposure to PTMCs activates the transcription factor NF-kB and the mitogen-activated protein kinase pathway. This study represents the first report indicating that testicular PTMCs generate MCP-1 upon direct exposure to MEHP via the interleukin-1 signaling pathway.

**Fig. 10.**
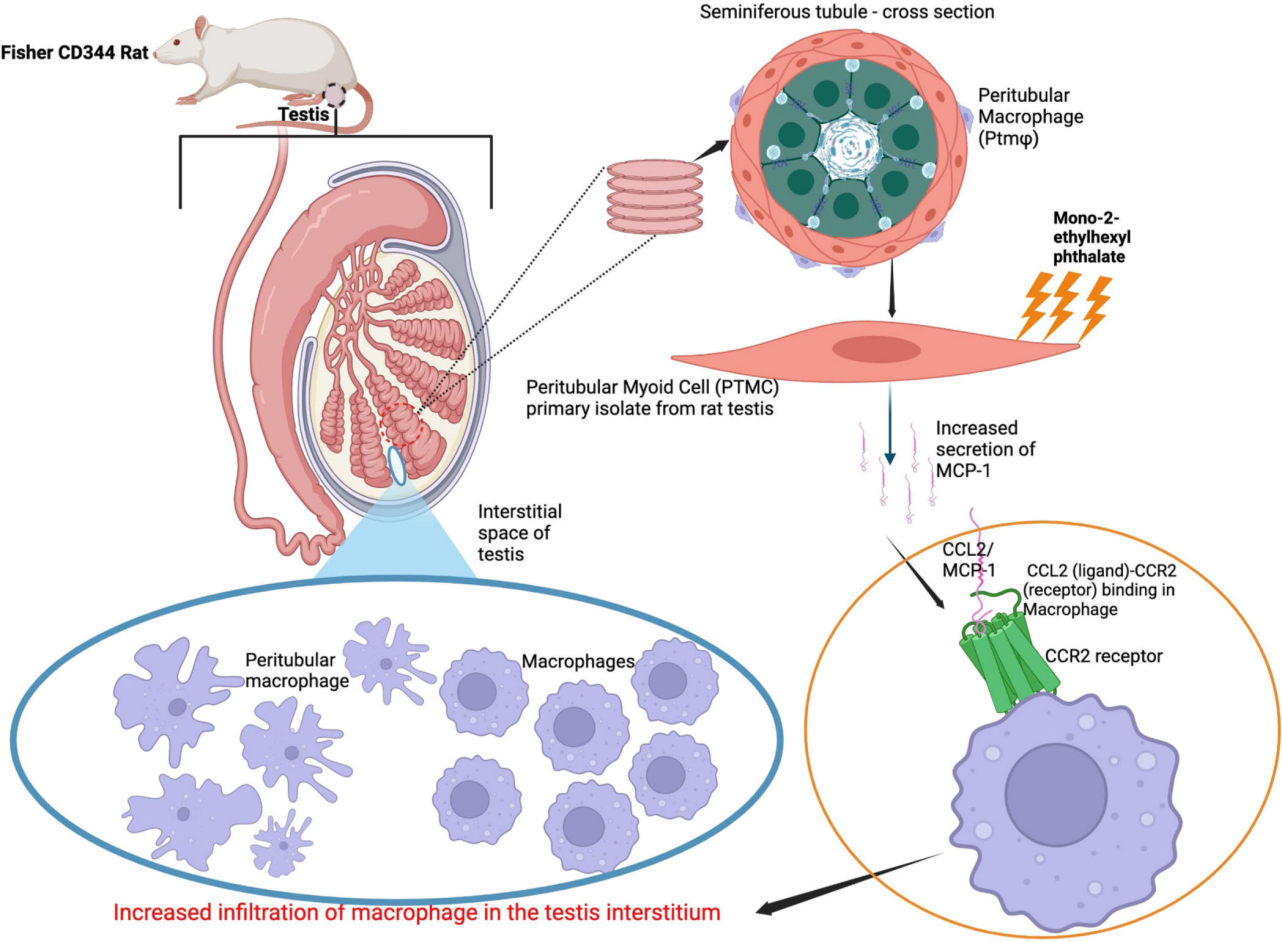
Graphical model demonstrating the role of MCP-1 secreted by PTMCs, followed by recruitment and activation of macrophages in the testis microenvironment in response to MEHP exposure.

PTMCs are a crucial structural component of seminiferous tubules and play a significant role in spermatogenesis, as well as in the functionality of other testicular cells (Maekawa et al., 1996; Zhou et al., 2019). The number of layers of PTMCs surrounding the ST varies by species, with a single layer in rodents and multiple layers in humans and non-human primates (Maekawa et al., 1996; Schmid et al., 2018). These cells not only facilitate sperm transport but also help maintain the SSC niche through their immunologically active secretions (Schmid et al., 2018). PTMCs secrete various extracellular matrix proteins, including fibronectin, collagens, SPARC, and fibrillin (Flenkenthaler et al., 2014), along with growth factors that influence Sertoli cell functions (Maekawa et al., 1996). They regulate spermatogenesis through several mechanisms, including androgen receptor (AR) (Welsh et al., 2009), P-Mod-S (Skinner et al., 1989), and Gata4 (Wang et al., 2018). The secretion of GDNF, a vital factor for SSC renewal (Chen et al., 2016, 2014; Spinnler et al., 2010), underscores the role of PTMCs in sustaining the SSC niche. Our previous study indicated that PTMCs produce MCP-1 in the peripubertal rat testis following MEHP treatment (Murphy et al., 2014). The number of peritubular macrophages (testis-specific macrophages) and both differentiating and undifferentiated spermatogonia were observed to increase simultaneously in the MEHP treatment group, accompanied by denser macrophage recruitment around the seminiferous tubules, where there was increased differentiation of SSCs (Gillette et al., 2021). These findings led us to conclude that peritubular macrophages play a distinct role in repairing toxicant-induced testis injury and restoring spermatogenesis. The finding of this study, combined with the evidence of our past studies indicating secretion of MCP-1 by PTMCs, followed by recruitment and activation of macrophages in the testis in response to toxicant exposure (Fig. 10), is an important piece of information. This is because testicular macrophages are known to be inherently immunosuppressive and help sustain the anti-inflammatory status in the testis microenvironment, thereby promoting spermatogonial cell proliferation and, eventually, male fertility (Piprek et al., 2024).

The cytokines interleukin-1β, TNF-α, and the chemokine MCP-1 in the acute high-dose MEHP-exposed testis microenvironment were confirmed in the interstitial fluid (IF) using a multiplex assay. A remarkable upregulation of IL-1β, TNF-α, and MCP-1 in the IF as early as six hours after exposure to MEHP indicates a pro-inflammatory cytokine response from the injured testicular tissue. MCP-1 is a primary chemokine of the testis (Aubry et al., 2000) and is produced by PTMCs in MEHP-exposed rats (Murphy et al., 2014). Our observation slightly differs from earlier reports where MCP-1 changed significantly after 12 hours of MEHP exposure (Aubry et al., 2000) and at a higher level. The difference in MCP-1 levels may be due to variations in samples, such as frozen tissue extracts and the dose of MEHP (1 g/kg). Additionally, the earlier study did not include the six-hour exposure time point. The descent increases in IL-1β, TNF-α, and MCP-1 after six hours of acute high-dose MEHP exposure in the testis microenvironment, suggesting two possibilities. First, acute high-dose MEHP exposure may cause physical or functional disruption of the blood-testis barrier (BTB) in prepubertal rats at an earlier time point than previously reported (Tiwary and Richburg, 2024). Second, these cytokines and chemokines may be produced by other cells, not by the cells in the ad-luminal space of the seminiferous tubules (ST), which require a compromised BTB to leak out.

We investigated a signal transduction pathway and established a mechanistic basis for MCP-1 secretion by PTMCs exposed to MEHP. Our previous study served as a guiding cue for MEHP exposure-associated MCP-1 production by PTMCs (Murphy et al., 2014). Compared to other cell types in the testes, PTMCs have been relatively overlooked; thus, their role in male fertility or infertility remains a potential area for future research. The PCA plot revealed that the concentration of MEHP (PC1) and the duration of exposure (PC2) induce differences in transcriptomes. Specifically, distinct changes were noted for the 200 μM MEHP exposure for 48 hours compared to a similar treatment group with a 12-hour exposure. This 48-hour exposure timeframe is supported by our prior publication, which observed more than a six-fold increase in the population of macrophages after 48 hours of exposure to MEHP (Gillette et al., 2021). Additionally, a notable influx of monocytes into the testis after 48 hours of MEHP exposure—more pronounced in younger animals, higher in rats than in mice, and occurring in a dose-dependent manner—further supports this finding (Murphy et al., 2014). The results from heatmaps and clustering analyses further illustrate changes in transcriptomes in PTMCs exposed to 200 μM MEHP for 48 hours. We identified numerous differentially expressed genes, including those related to interleukin-1 signaling pathways such as Map3k7/Tak1, Mapkapk2/Mk2, Nlrp3, and others (See Supplementary Fig. 4A&B). Our findings align with prior studies demonstrating significant upregulation of Rela protein and mRNA expression upon exposure to MEHP (Gupta et al., 2010; Rasoulpour and Boekelheide, 2005; Wang et al., 2017). The Nucleotide-binding domain, leucine-rich repeat-containing protein 3 (Nlrp3) gene is activated by various stressors, including reactive oxygen species (ROS), reactive nitrogen species (RNS), ionic imbalance, dysfunctional cell organelles, and lysosomal damage, responding through inflammasome formation and pro-inflammatory cytokine IL-1 production, either to combat infection or repair damaged tissue (Kelley et al., 2019). Previous studies have shown that MEHP exposure leads to Nlrp3 inflammasome activation (Park et al., 2019), and our findings are consistent with this.

We observed significant upregulation of Mapkapk2 or Mk2 in the treatment group. Its expression in smooth muscle cells (Hedges et al., 1999) and its roles in the inflammatory response have been well established (Kotlyarov et al., 1999). MK2 is a crucial substrate of p38MAPK that influences the production of inflammatory cytokines, cell signaling, transcript stability, and other vital cellular processes. It participates in the post-translational regulation of cytokines, chemokines, and pro-inflammatory factors through RNA-binding proteins (Gaestel, 2006; Soni et al., 2019). Scientific evidence indicates that a lack of MK2-mediated mRNA stabilization results in reduced or diminished expression of genes involved in inflammatory responses (Gaestel et al., 2009). Similarly, Tak1 (Map3k7), which regulates cytokine production as a central component of the canonical IL-1 signaling pathway, is well recognized (Gaestel et al., 2009). A notable upregulation of Tak1 expression in the 48-hour 200 μM MEHP treatment group but not in the corresponding control or 12-hour exposure group suggests that the IL-1 signaling pathway drives MCP-1 production. These results were further validated by the quantitative real-time PCR experiment used to assess the expressions of selected IL-1 signaling pathway genes.

GO and GSEA analyses were conducted to gain insight into the transcription factors and signaling pathways associated with differentially expressed genes, utilizing databases such as REACTOME, KEGG, WP, HP, and others. The most significantly enriched GO: BPs identified in this study included the positive regulation of NF-kB transcription factor activity, innate immune response, positive regulation of cytokine production involved in the immune response, cytokine production related to the immune response, regulation of cytokine production, positive regulation of the production of molecular mediators of immune response, and immune response-activating signaling pathways. These GO terms clearly indicate NF-kB activation, followed by cytokine and chemokine production, which drives the innate immune response. Notably, MEHP exposure leading to an immune response in the testis has been frequently recognized (Fang et al., 2024b; Gillette et al., 2021; Rasoulpour and Boekelheide, 2005; Voss et al., 2018). The Reactome pathway enrichment analysis revealed significant enrichment in the MyD88: MAL (TIRAP) and TLR4 signaling pathways. These pathways involve MyD88, an adaptor protein that links IL-1R or TLR to the IL-1R-associated kinase (IRAK) family, ultimately activating the transcription factor NF-kB, mitogen-activated protein kinases (MAPK), and activator protein 1 (AP-1) (Deguine and Barton, 2014). Similarly, ERK/MAPK targets, as well as MAPK targets/Nuclear events, were significantly enriched, highlighting the crucial roles that MAP kinases play in activating transcription factors NF-kB to enhance MCP-1 gene expression (Plotnikov et al., 2011; Wolter et al., 2008). More specifically, the transcription factor NF-kB functions alongside ERKs to regulate ATF2:cJun and AP-1, with AP-1 responsible for producing IL-1 target genes, including MCP-1, in mammalian cells (Gaestel et al., 2009; Plotnikov et al., 2011; Weber et al., 2010; Wolter et al., 2008).

In contrast to the results of GO enrichment analysis, those from gene set enrichment analysis (GSEA) are more quantitative and definitive. Our GSEA findings reveal significant enrichment of the interleukin-1 signaling pathway in PTMCs following exposure to MEHP. The IL-1 signaling pathway is a well-established mechanism for chemokine secretion (MCP-1/CCL2) in other cell types (Gaestel et al., 2009; Weber et al., 2010). Furthermore, our GSEA results highlight several other relevant pathways that are significantly enriched; including regulation of Slits and Robos, cellular response to chemical stress, cellular response to starvation, mitochondrial gene module, the non-canonical NF-kB signaling pathway, and others (Supplementary Fig. 5). Our observation is well aligned with the previous studies that have reported oxidative stress, mitochondrial dysfunction, NF-kB activation, and testicular cell death as consequences of MEHP exposure (Kasahara et al., 2002; Onorato et al., 2008; Rasoulpour and Boekelheide, 2005; Richburg, 2006; Richburg and Boekelheide, 1996).

The GSEA results indicate that the significant enrichment of the regulation of the expression of Slits and Robos. Recent scientific evidence regarding the activity of the Slit2 neuro-repellent protein indicates that it promotes macrophage recruitment and polarization while inhibiting T lymphocyte infiltration in a mouse model (Geraldo et al., 2021). Furthermore, Slit2-induced inhibition of macrophage dispersal or promotion of their clustering via the Slit2-Robo1-Myo9b-RhoA signaling pathway has been reported (Bhosle et al., 2020). In contrast, Slit2 has been shown to prevent the mobility of chemokines and vascular smooth muscle cells through its cognate receptor Roundabout 1 (Robo-1) (Mukovozov et al., 2015). Considering our earlier findings, MEHP treatment leads to a significant increase in the number of immune cells, including peritubular macrophages, in the testicular environment (Gillette et al., 2021; Murphy et al., 2014; Voss et al., 2018); presumably, both MCP-1 and the Slit2/Robo signaling pathway regulate immune cell responses in the testis. Indeed, future studies should focus on the precise role of Slit/Robo signaling in MEHP-treated testes.

To further confirm our findings from the transcriptomics experiment, we conducted a proteomics analysis on PTMC lysates. This analysis revealed numerous proteins associated with the IL-1 signaling pathway, including proteasomes, ubiquitins, Cullin 1, and Mitogen-activated protein kinase 1 (Mapk1 or ERK2), all of which respond to MEHP treatment. Proteasomes efficiently facilitate targeted protein degradation in association with ubiquitin, as the binding of ubiquitin serves as a signal for degradation. The ubiquitin-proteasome system regulates various cellular processes, including signal transduction, protein quality control, and immune responses (Tanaka, 2009; Wang and Maldonado, 2006). The critical roles of proteasomes and ubiquitins in the IL-1 signaling pathway are well documented (Weber et al., 2010), and as the ubiquitin-proteasome system is functionalized, its upregulation is anticipated. Cullin 1 acts as a central scaffold protein within the SCF complex (SKP1-CUL1-F-box protein), which works with E3 ubiquitin ligase to ubiquitinate proteins during signal transduction and transcription (Kipreos et al., 1996; Vijayakumar et al., 2022). Importantly, the significant role of ERK signaling in MCP-1 production has been previously established (Oh et al., 2001).

The protein network of the IL-1 signaling pathway, supplemented with differential gene expression data from RNA sequencing analysis, effectively visualizes the genes and their associated proteins within this pathway. This observation aligns with findings from proteomics, which identified numerous proteins in the IL-1 signaling pathway that are upregulated following MEHP exposure. Gene expression analysis conducted via qRT-PCR also revealed significant upregulation of selected genes within the IL-1 signaling pathway. Furthermore, the results from the immunofluorescence experiment on testis sections strongly correlate with findings from other experimental techniques.

## 5. Conclusion

In summary, for the first time, we report that primary PTMC isolates from Fisher rats produce MCP-1 upon direct MEHP exposure via the IL-1 signaling pathway, both in vitro and in vivo. MEHP exposure, linked to chemical or oxidative stress, activates NF-kB and the MAP kinase pathway, leading to MCP-1 production. Our proteomic analysis and quantitative PCR results further support this novel discovery, demonstrating that direct MEHP exposure alone is sufficient to induce MCP-1 production by PTMCs, independent of factors from other Sertoli or germ cells. This new understanding of MCP-1 regulation by PTMCs is particularly noteworthy, as it underscores their crucial role in modulating immune responses within the SSC niche. This finding advances our knowledge of PTMCs’ function and opens new avenues for research into immune regulation and potential therapeutic interventions targeting the SSC niche.

## Supplementary Material

Combined suppl Figs with legends

## Figures and Tables

**Table 1.** qRT-PCR primers

SN	Target genes	Forward primer	Reverse primer
1	*Acta2*	CATCCGACCTTGCTAACGGA	GTCCAGAGCGACATAGCACA
2	*Hsd17b3*	TCCAGGTGCTGACCCCTTAT	CAAACTCATCGGCGGTCTTG
3	*Sox9*	GAAGTCGGTGAAGAATGGGC	CCTGAGATTGCCCGGAGTG
4	*Gapdh*	GCCAGTAGACTCCACGACA	GCAAGTTCAACGGACAAG
5	*Vasa*	CAGAGGGAGCGAGAACAAG	TGCCAGTATTTCCACAACG

6	*Il-1b*	TTGAGTCTGCACAGTTCCCC	GTCCTGGGGAAGGCATTAGG
7	*Il-1r1*	CCTCTGCCTCTTGACGATGG	TGGTATGTGTAGGACGTGCG
8	*Il-1r2*	GCTGGAAAGACGTATGGGCT	TTTGGTTTGGGCTGGAAGGG
9	*Myd88*	CCTGGTTCTGGACCCGTCTT	CTGGTTGCTCAGGCCAGTCA
10	*Traf-6*	ATCTCGGAGTGCTGCGTGTA	TCGCTTGAAGACTGGCTGGA
11	*Tlr-4*	ACAGGGCACAAGGAAGTAGC	GTTCTCACTGGGCCTTAGCC
12	*Nlrp3*	GACTTCTGCACCCCGACTGT	ACAGAGCGTCACCACACACA
13	*Caspase-1*	GACCGAGTGGTTCCCTCAAGT	GCAAGACGTGTACGAGTGGGT
14	*Mcp-1*	AGCCAACTCTCACTGAAGCC	AACTGTGAACAACAGGCCCA

**Table 2.** Il-1 signaling pathway proteins

				Exclusively unique peptide count	

Identified Proteins	Accession number	Alternate ID	Mol. Wt. kDa	Control	MEHP 200 µM

Proteasome subunit alpha type-6	P60901	PSMA6	27	6	8
Proteasome subunit alpha type-3	P18422	PSMA3	28	8	9
Proteasome subunit alpha type-4	P21670	PSMA4	29	6	7
Proteasome subunit beta type-4	P34067	PSMB4	29	4	5
Proteasome activator subunit 4	A0A0G2K9R9	PSME4	211	7	8
Proteasome subunit alpha type-5	P34064	PSMA5	26	3	4
26S proteasome regulatory subunit 4	P62193	PSMC1	49	2	4
26S proteasome non-ATPase regulatory subunit 11	F1LMZ8	PSMD11	47	3	4
26S proteasome non-ATPase regulatory subunit 1	O88761	PSMD1	106	1	4
26S proteasome regulatory subunit 7	Q63347	PSMC2	49	2	3
Proteasome subunit beta type-10	Q4KM35	PSMB10	29	1	2
26S proteasome regulatory subunit 8	P62198	PSMC5	46	2	4
26S proteasome regulatory subunit 6B	Q63570	PSMC4	47	0	2
26S proteasome non-ATPase regulatory subunit 13	B0BN93	PSMD13	43	0	1
Proteasome 26S subunit, ATPase 6	A0A8I5ZU65	PSMC6	44	0	1
26S proteasome non-ATPase regulatory subunit 5	G3V8G2	PSMD5	56	0	2
26S proteasome regulatory subunit 6A	Q63569	PSMC3	49	0	1
26S proteasome non-ATPase regulatory subunit 8	F1LMQ3	PSMD8	40	0	1
Inhibitor of nuclear factor kappa-B kinase-interacting protein	Q5EAJ6	IKBIP	42	1	2
Fos-related antigen 2	P51145			0	1
E2 ubiquitin-conjugating enzyme	A0A8I6A356	UBE2K	31	5	7
Ubiquitin-conjugating enzyme E2H	M0R4P9	UBE2H	21	2	3
Ubiquitin-like modifier-activating enzyme 1	Q5U300	UBA1	118	31	32
Ubiquitin-60S ribosomal protein L40	P62986	UBA52	15	2	3
Ubiquitin associated protein 2-like	A0A8I6ACU2	UBAP2L	115	0	2
NEDD8-activating enzyme E1 catalytic subunit	Q99MI7	UBA3	52	0	1
Ubiquitin-like modifier-activating enzyme 5	Q5M7A4	UBA5	45	0	1
Culin 1	A0A8I5Y0H0	CUL1	90	0	1
Reticulocalbin 1	D3ZUB0	RCN1	38	0	1
Mitogen-activated protein kinase 1	P63086	MAPK1	41	9	10
Mitogen-activated protein kinase 3	P21708	MAPK3	43	4	5
MAP7 domain containing 1	A0A8I5ZM56	MAP7D1	94	1	2

## Data Availability

Data will be made available on request.

## Appendix A. Supporting information

Supplementary data associated with this article can be found in the online version at doi:10.1016/j.tox.2025.154118.

## Abbreviations:

PTMCs: Peritubular myoid cells
MEHP: Mono-2-ethylhexyl phthalate
DEHP: Di2-ethylhexyl phthalate
STs: Seminiferous tubules
SSC: Spermatogonial stem cell
MCP-1: Monocyte chemotactic protein 1
IL-1β: Interleukin-1beta
LIF: Leukemia inhibitory factor
GDNF: Glial cell line-derived neurotrophic factor
BTB: Blood-testis barrier
TLRs: Toll-like receptors
TNF-α: Tumor Necrosis Factor-alpha
CdCl_2_: Cadmium chloride
MAA: Methoxy acetic acid
DSCPs: Damaged spermatocytes/spermatogonial cell particles
PND: Postnatal day
DAPI, 4’: 6-diamidino-2-phenylindole
IF: Interstitial fluid
RT: Room temperature
TMM: Trimmed Mean of M-values
GO: Gene Ontology
GSEA: Gene Set Enrichment Analysis
DEGs: Differentially Expressed Genes
PCA: Principal component analysis
MF: Molecular Functions
CC: Cellular Compartments
BP: Biological Processes
KEGG: Kyoto Encyclopedia of Genes and Genomes
TF: Transcription factor
WP: Wiki Pathways
NF-kB: Nuclear Factor Kappa B
NES: Normalized enrichment scores
Nlrp3: Nucleotide-binding domain, leucine-rich repeat-containing protein 3
Il-1r1: Interleukin-1 receptor type 1 1
