# Supplementary material for: Peritubular myoid cells of the testis produce monocyte chemotactic protein 1 upon direct exposure to Mono-(2-Ethylhexyl) phthalate through the IL-1 signaling pathway
Source: Toxicology. Author manuscript; Available in PMC 2025 Nov 24. (PMC12643028; doi:10.1016/j.tox.2025.154118)
Supplement: Combined suppl Figs with legends [file NIHMS2109641-supplement-Combined_suppl_Figs_with_legends.pdf]

Supplementary Figure 1A

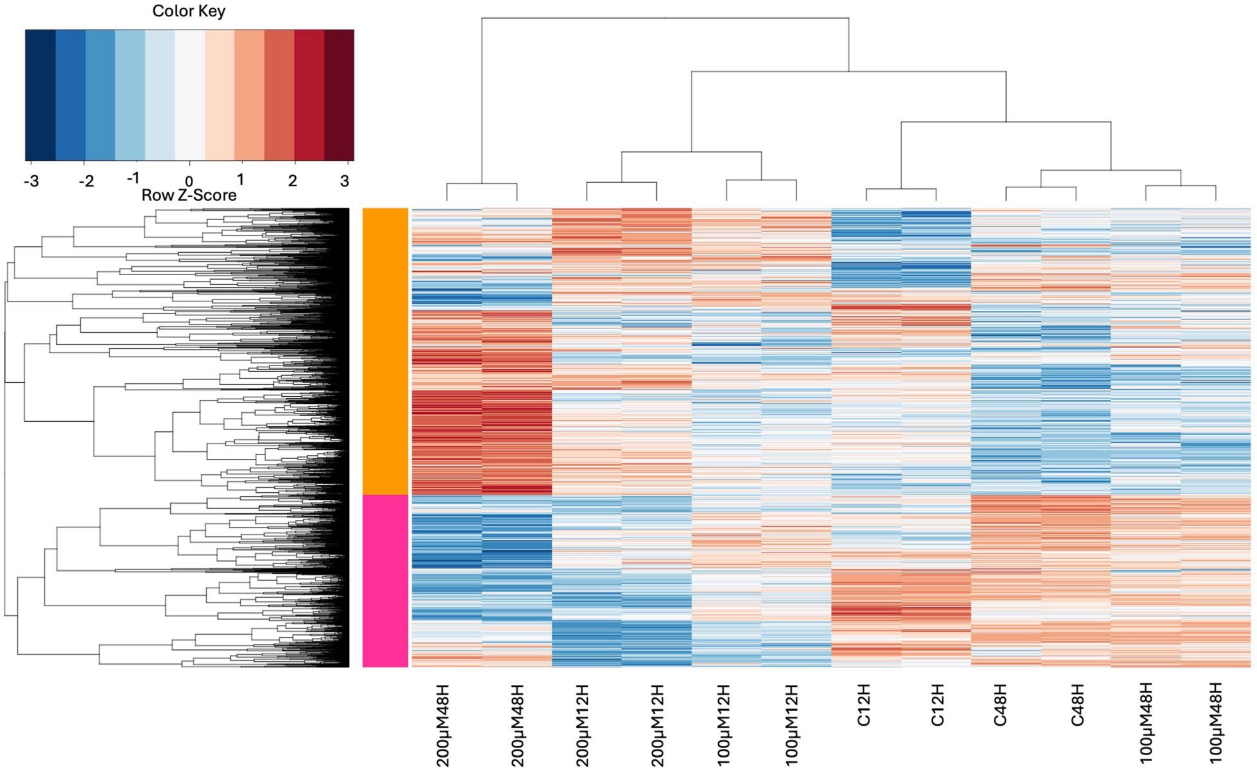

Supplementary Figure 1A. Heat map demonstrating modules of upregulated and downregulated genes in control, 100µM, and 200µM MEHP exposure for 12 and 48 hours. The Z-score bar indicates a relative expression +/- standard deviation from the mean, and the False discovery rate (FDR) is less than 0.05. DEGs were selected using p-value = 0.05 and log base2 fold change equal to 0.5.

Supplementary Figure 1B

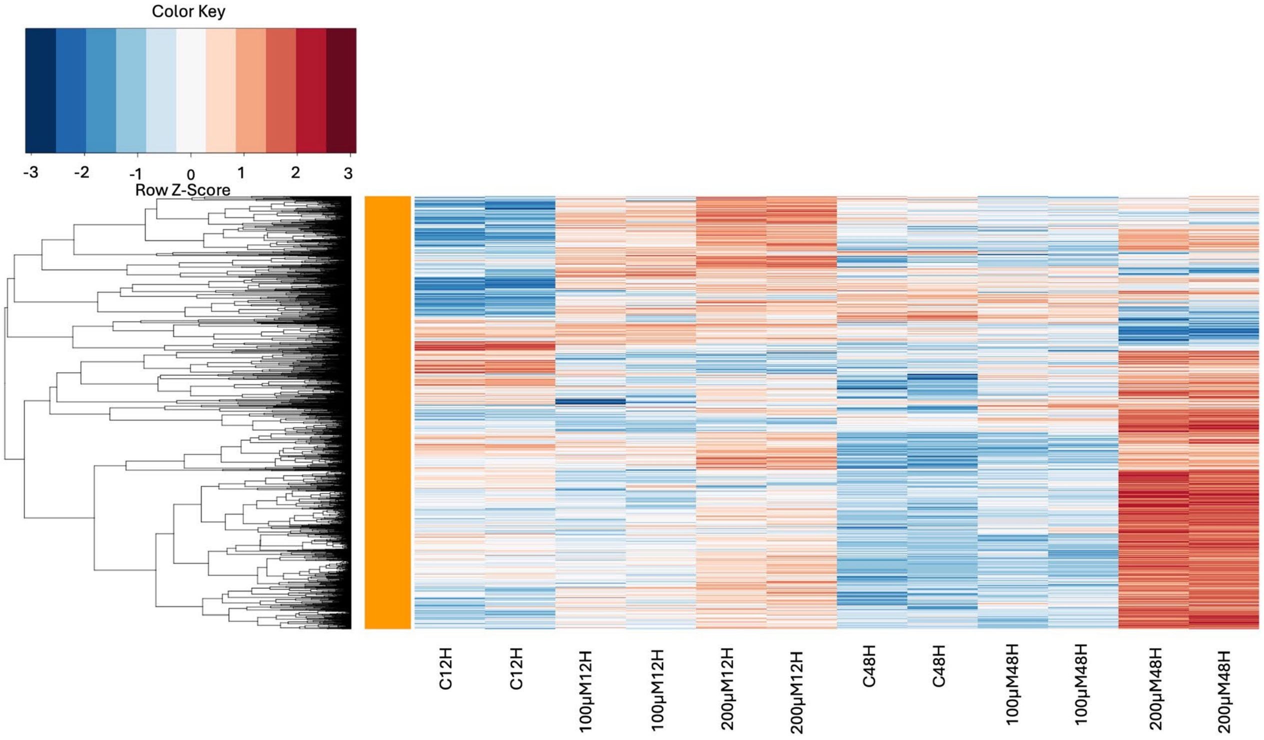

Supplementary Figure 1B. Heat map demonstrating a module of upregulated genes in control, 100μM, and 200μM MEHP exposure for 12 and 48 hours. The Z-score bar indicates a relative expression +/- standard deviation from the mean, and the False discovery rate (FDR) is less than 0.05. DEGs were selected using p-value = 0.05 and log base2 fold change equal to 0.5.

Supplementary Figure 1C

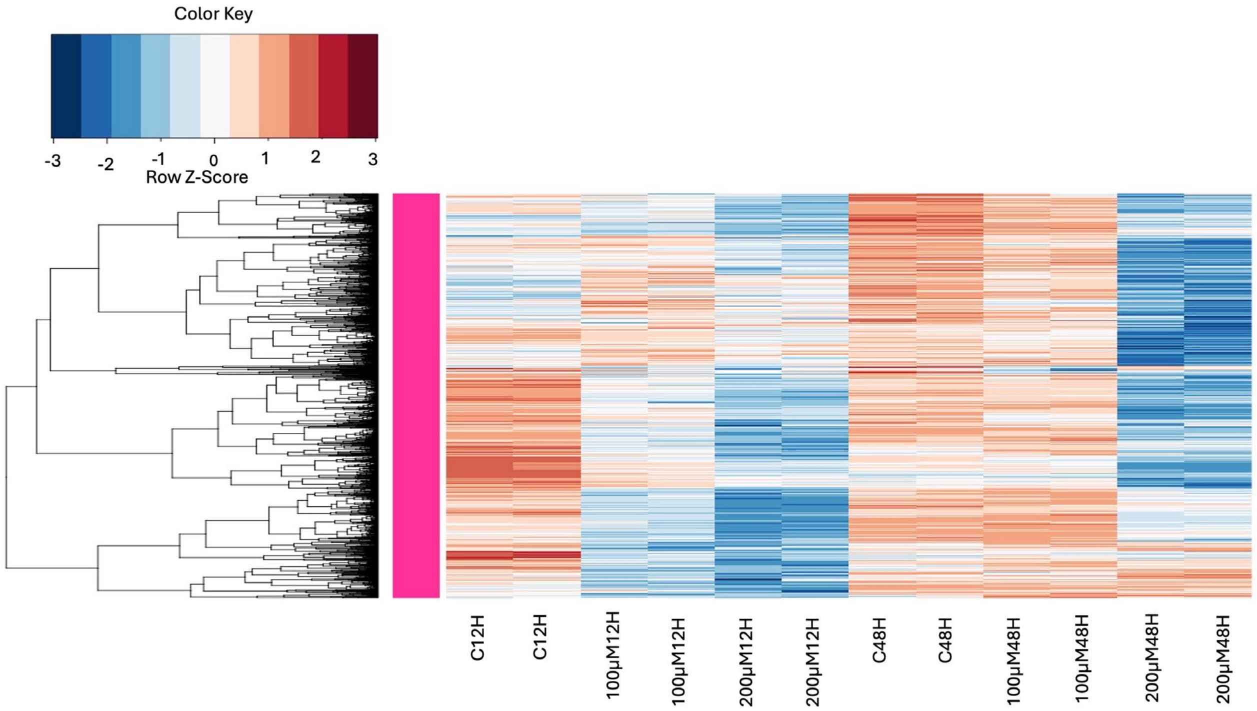

Supplementary Figure 1C. Heat map demonstrating a module of downregulated genes in control, 100μM, and 200μM MEHP exposure for 12 and 48 hours. The Z-score bar indicates a relative expression +/- standard deviation from the mean, and the False discovery rate (FDR) is less than 0.05. DEGs were selected using p-value = 0.05 and log base2 fold change equal to 0.5.

Supplementary Figure 2

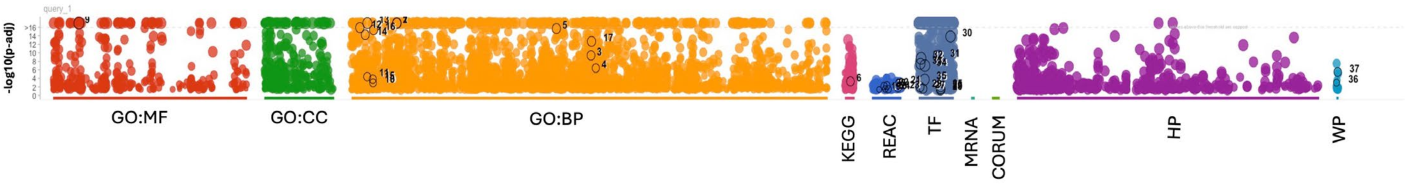

id	source	term_id	term_name	term_size	p_value
1	GO:BP	GO:0006954	inflammatory response	431	1.7e-18
2	GO:BP	GO:0006955	immune response	940	5.0e-25
3	GO:BP	GO:0050727	regulation of inflammatory response	232	4.0e-10
4	GO:BP	GO:0051092	positive regulation of NF-kappaB transcription factor activity	104	3.8e-07
5	GO:BP	GO:0045087	innate immune response	430	1.9e-16
6	KEGG	KEGG:04621	NOD-like receptor signaling pathway	173	5.0e-04
9	GO:MF	GO:0005515	protein binding	6872	3.2e-269
10	GO:BP	GO:0002720	positive regulation of cytokine production involved in immune response	58	1.1e-03
11	GO:BP	GO:0002367	cytokine production involved in immune response	86	3.9e-05
12	GO:BP	GO:0001817	regulation of cytokine production	500	1.2e-16
13	GO:BP	GO:0002376	immune system process	1521	1.6e-50
14	GO:BP	GO:0002253	activation of immune response	314	5.4e-15
15	GO:BP	GO:0002702	positive regulation of production of molecular mediator of immune response	95	1.6e-04
16	GO:BP	GO:0002757	immune response-activating signaling pathway	272	4.1e-16
17	GO:BP	GO:0050778	positive regulation of immune response	436	2.0e-13
18	REAC	REAC:R-RNO-450282	MAPK targets/ Nuclear events mediated by MAP kinases	18	3.1e-02
19	REAC	REAC:R-RNO-198753	ERK/MAPK targets	12	4.2e-02

id	source	term_id	term_name	term_size	p_value
20	REAC	REAC:R-RNO-448424	Interleukin-17 signaling	36	6.3e-03
21	REAC	REAC:R-RNO-166016	Toll Like Receptor 4 (TLR4) Cascade	64	1.4e-03
22	REAC	REAC:R-RNO-166058	MyD88:MAL(TIRAP) cascade initiated on plasma membrane	57	5.2e-03
23	REAC	REAC:R-RNO-75893	TNF signaling	40	1.9e-02
24	REAC	REAC:R-RNO-168643	Nucleotide-binding domain, leucine rich repeat containing receptor (NLR) signaling pathways	34	3.5e-02
25	TF	TF:M08891_1	Factor: NFKAPPAB; motif: NNGGGANTTCCCN; match class: 1	122	1.2e-02
26	TF	TF:M00208_1	Factor: NF-kappaB; motif: NGGGACTTTCCA; match class: 1	440	2.7e-02
27	TF	TF:M07414_1	Factor: C/EBPdelta; motif: RTTGCGWAAY; match class: 1	176	2.7e-02
28	TF	TF:M00054_1	Factor: NF-kappaB; motif: GGGAMTTYCC; match class: 1	620	5.0e-02
29	TF	TF:M07349_1	Factor: AP-2gamma; motif: NNNNWGCCYNCRGSCN; match class: 1	301	9.7e-03
30	TF	TF:M00115_1	Factor: Tax/CREB; motif: RTGACGCATAYCCCC; match class: 1	2690	1.4e-14
31	TF	TF:M00279	Factor: MIF-1; motif: NNGTTGCWWGGYAACNGS	13690	6.9e-10
32	TF	TF:M00691	Factor: ATF-1; motif: CYYTGACGTCA	5311	1.9e-09
33	TF	TF:M01860_1	Factor: AP-4; motif: NCAGCTGYNGNCN; match class: 1	1416	3.6e-08
34	TF	TF:M00801	Factor: CREB; motif: CGTCAN	4819	9.7e-08
35	TF	TF:M00041_1	Factor: ATF2:c-Jun; motif: TGACGTYA; match class: 1	1541	1.9e-04
36	WP	WP:WP294	p38 Mapk signaling pathway	28	1.0e-03
37	WP	WP:WP457	TNF-alpha NF-kB signaling pathway	143	3.1e-06

g:Profiler (biit.cs.ut.ee/gprofiler)

Supplementary Figure 2. Functional enrichment analysis (GO enrichment) of the ranked list of genes. GO enrichment was performed to get insight into specific biological processes (BP), molecular functions (MF), cellular compartments (CC), pathways, and transcription factors associated with the differentially expressed genes in the group 200μM MEHP exposure for 48 hours.

Supplementary Figure 3

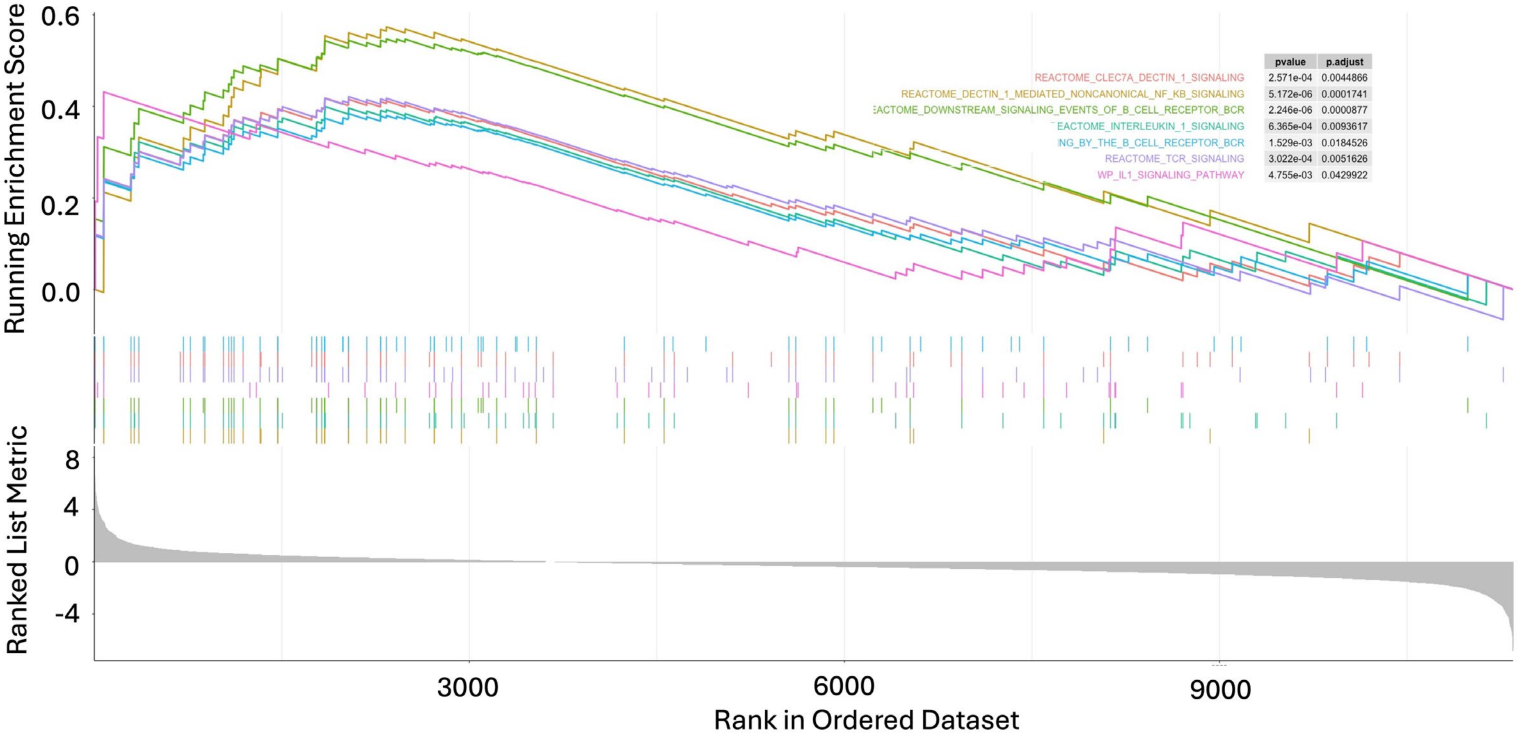

Supplementary Figure 3. Functional enrichment analysis using Gene Set Enrichment Analysis (GSEA) to identify a specific pathway enrichment status. Reactome CLECTA Dectin 1 signaling, Reactome Dectin 1 mediated non-canonical NF-kB signaling, Reactome downstream signaling events of B Cell Receptor, Reactome IL-1 Signaling pathway, Reactome signaling by the B Cell receptor, Reactome TCR signaling, WikiPathway IL-1 Signaling Pathway were significantly enriched in the group 200 µM MEHP exposed for 48 hours.

Supplementary Figure 4

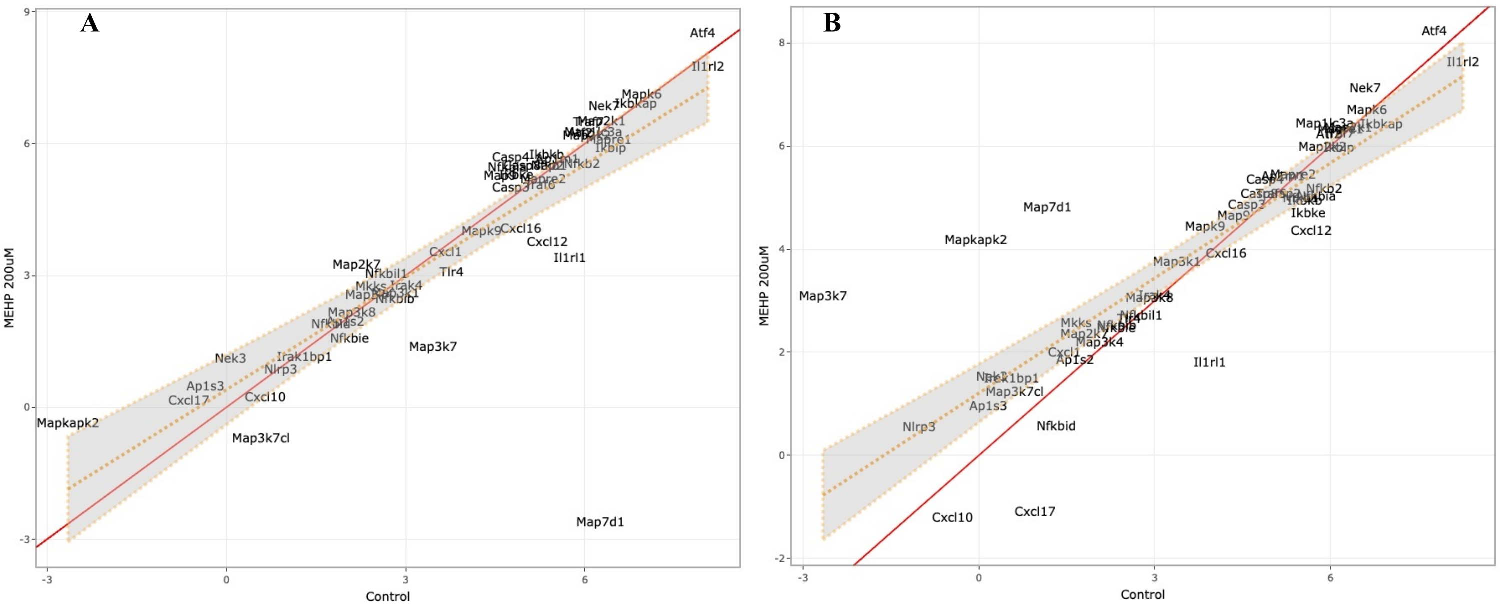

Supplementary Figure 4. Average expression of IL-1 signaling pathway genes within the control group vs 200 μM MEHP exposure group. **A**, 12 hours. **B,** 48 hours.

Supplementary Figure 5

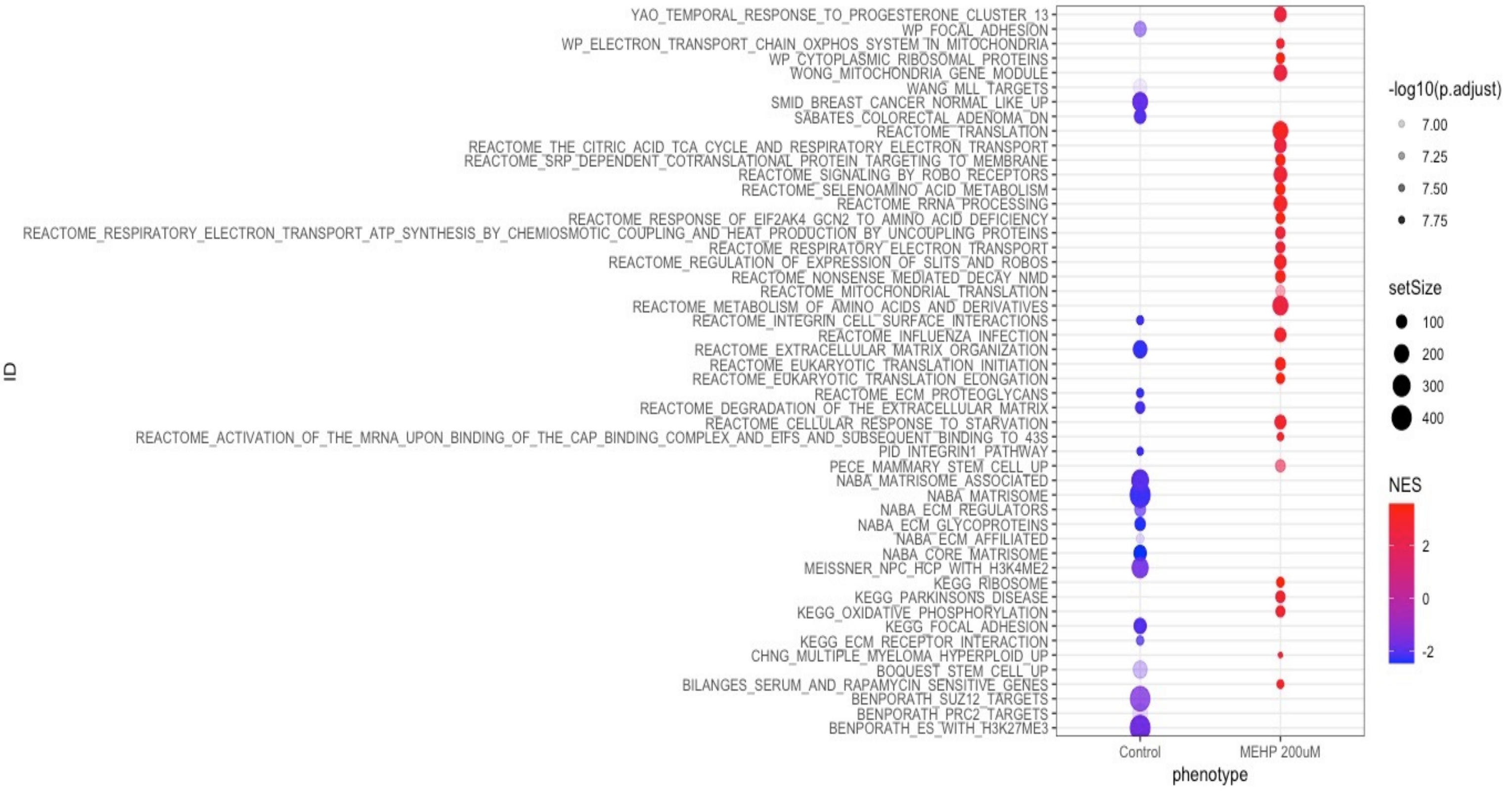

Supplementary Figure 5. Bubble plot demonstrating a significant enrichment of the top 50 pathways. Functional enrichment analysis was performed using Gene Set Enrichment Analysis (GSEA) to identify a specific pathway enrichment status in the group 200μM MEHP exposure for 48 hours.
